# A qualitative study exploring depressed participants’ experiences of receiving Augmented Depression Therapy (ADepT)

**DOI:** 10.1136/bmjopen-2024-088726

**Published:** 2025-02-05

**Authors:** Kalliopi Demetriou, Emily Widnall, Laura Warbrick, Nigel Reed, Katie Marchant, Nicole Geschwind, Rebecca Watson, Isabella Magner-Parsons, Rachel Barter, Kim A Wright, Barney Dunn

**Affiliations:** 1 University of Edinburgh, Edinburgh, UK; 2 Bristol Medical School, University of Bristol, Bristol, UK; 3 Mood Disorders Centre, University of Exeter, Exeter, UK; 4 Department of Clinical Psychological Science, Maastricht University, Maastricht, The Netherlands; 5 University of Oxford, Oxford, UK; 6 Somerset Foundation Trust NHS Talking Therapies Service, Taunton, UK

**Keywords:** depression & mood disorders, psychosocial intervention, qualitative research

## Abstract

**Objectives:**

The current study aimed to explore participants’ views on the acceptability, impact and mechanisms of change of Augmented Depression Therapy (ADepT), a novel wellbeing-focused and recovery-oriented psychological therapy for depression.

**Design:**

A semi-structured qualitative interview design was used, with data analysed using the framework approach.

**Participants:**

20 participants with anhedonic depression who had received up to 20 sessions of ADepT, sampled from a pilot randomised controlled trial of ADepT versus Cognitive Behavioural Therapy (CBT).

**Setting:**

A primary care psychological therapy clinic in Devon, UK, with interviews occurring between May 2018 and February 2020.

**Results:**

Participants found the wellbeing focus of ADepT acceptable. Helpful aspects of therapy were a positive therapeutic bond, the structure and flow of therapy scaffolding the learning journey, the tools and techniques of therapy helping building wellbeing and booster sessions supporting long-term recovery. Negative aspects for some participants were therapy feeling too intense and triggering feelings of failure. Participants reported significant positive impacts of treatment on wellbeing, functioning and hope. Perceived mechanisms of change were reorienting to the positive, engaging with valued goals, taking a proactive life stance, gaining confidence and motivation for change, breaking down tasks into small steps, cultivating self-care and self-compassion, enhancing help seeking and interpersonal effectiveness, changing the relationship to depression, and rediscovering the self beyond depression.

**Conclusions:**

Findings suggest that the wellbeing focus of ADepT is acceptable and leads to positive impacts, supports the logic model underpinning the intervention, and warrants continuation to a definitive trial.

**Trial registration number:**

ISRCTN85278228.

**STRENGTHS AND LIMITATIONS OF THIS STUDY**

- The study explored participants’ experience of Augmented Depression Therapy (ADepT) using the Framework Method, enabling an in-depth qualitative process evaluation of treatment acceptability, impacts, mechanisms of treatment, and contextual modifying factors.
- Reflecting the positive results of the pilot trial, the interviews were predominantly conducted with participants who fully engaged with and benefited from treatment, and we have limited sampling of participants who experienced less positive outcomes.
- Representing the demographics of the Devon region from which the trial recruited, the sample available for interview was predominantly of White British ethnicity, and experiences of treatment may differ in other ethnic groups.
- There was a variable length of time between participants completing acute treatment and being interviewed (6–25 months), although there was no evidence that this impacted participants’ capacity to recall their experience of therapy or their views of ADepT.

## Introduction

 Depression is a prevalent, disabling and often chronic, relapsing and recurrent mental health condition that is a major contributor to worldwide disability and results in significant societal and economic costs.^1^,^4^ While effective psychological treatments for depression do exist, they are not optimised. Less than 50% of individuals treated with current gold-standard evidence-based therapies, such as Cognitive Behavioural Therapy (CBT), respond or fully remit,^5 6^ with more than half of them experiencing relapse within 2 years.^7^ This results in a sustained recovery rate of around 25%.

Alongside symptoms of depression, individuals often experience significant reductions in wellbeing (the capacity to experience pleasure, meaning and social connection) and deficits in everyday functioning, relative to their own levels before becoming depressed and to typical values in the general population.^8 9^ Reflecting the centrality of these wellbeing changes, participants’ lived experience accounts of depression emphasise an inability to experience positive emotions, feeling numb and empty, losing the capacity to act on the world, losing a sense of purpose and existential hope, a stagnation of the present and a sense of impossibility about a positive future.^10^ Restoration of wellbeing and functioning is seen as being critical to personal recovery from depression and yet often lags behind symptom relief.^11^,^13^ Wellbeing and functioning difficulties can persist in subsyndromal form in between acute episodes^9 14^ and confer vulnerability to recurrence/relapse and ongoing low mood.^15 16^ Current psychological therapies focus predominantly on reducing symptoms of depression but relatively neglect enhancing wellbeing and functioning,^8 17 18^ potentially contributing to their suboptimal long-term outcomes.^19^ There is a need to develop enhanced treatments that target both symptom relief *and* restoration of wellbeing and functioning to minimise the burden of illness associated with depression.^19^

A novel psychotherapy, Augmented Depression Therapy (ADepT), has therefore been developed to both reduce depression symptoms *and* enhance wellbeing and functioning, and shows potential to lead to enhanced treatment outcomes over the long term.^18 20^ ADepT was co-designed alongside experts by experience and clinicians, informed by the intervention mapping framework^21^ and Medical Research Council guidance for complex intervention development.^22^ While a range of other therapies are being developed to explicitly target positive mental health outcomes across clinical disorders, ADepT is relatively unique in its combined focus on both symptom relief and wellbeing enhancement (a hybrid approach) and its explicit focus on depression.^18 23^

**Figure 1:**
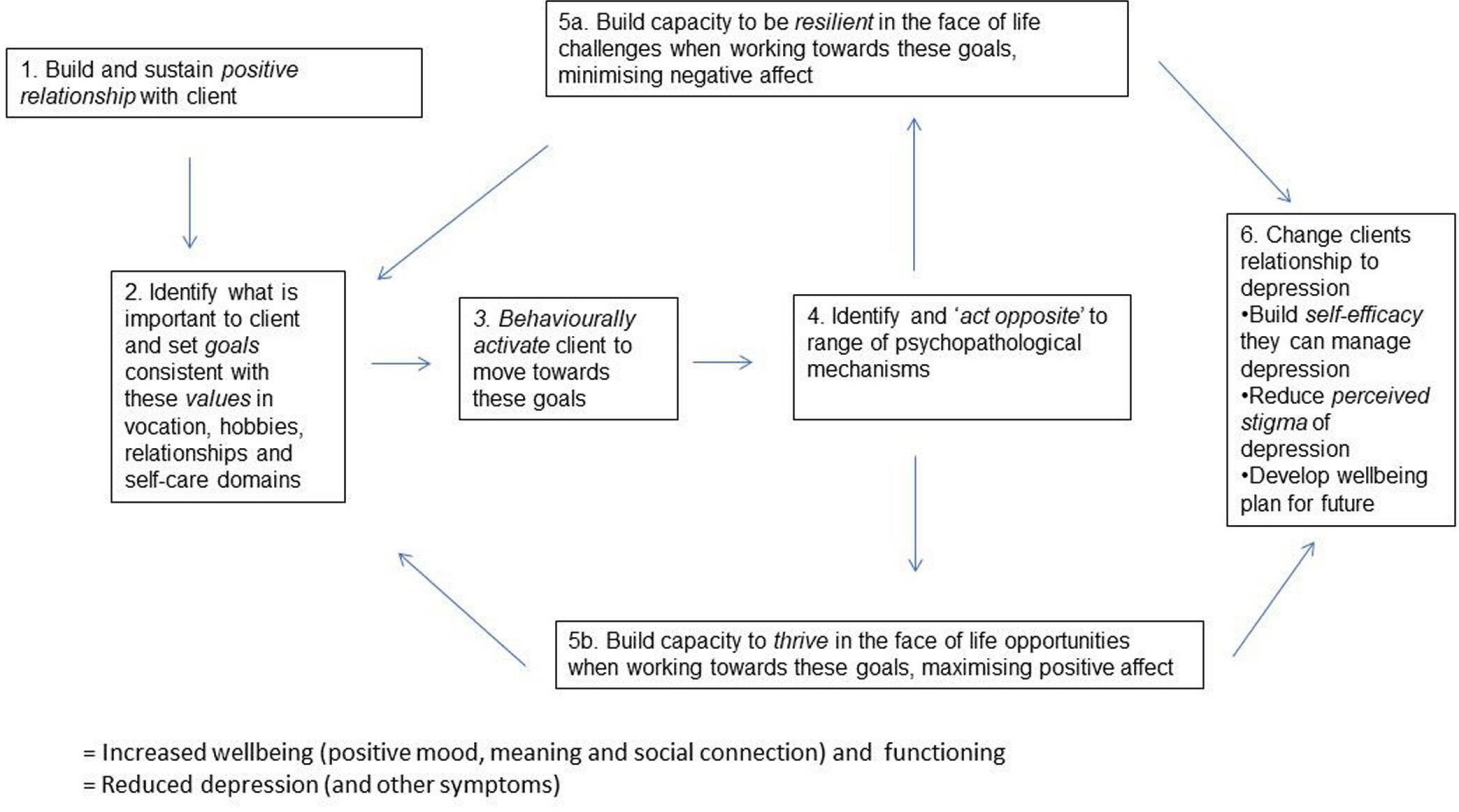
Logic model of augmented depression therapy intervention.

ADepT is a solution-focused, cognitively augmented, behavioural activation individual treatment approach (15 acute session and five booster sessions).^18 20^ Building wellbeing (the capacity to experience pleasure, meaning and social connection in life) and functional recovery is the primary focus, with depression conceptualised as patterns of thinking, feeling and behaving that serve as barriers to achieving this goal. Therapy involves identifying participants’ values in four key life domains (vocation, hobbies, relationships and self-care); behaviourally activating participants to work towards values-consistent goals in these domains; and noticing and overcoming barriers to being resilient (managing challenges to reduce negative affect) and thriving (taking opportunities to maximise positive affect). Therapists are trained to use a positively oriented, future-directed, solution-focused, warm and friendly interpersonal style that aims to attend to and ‘thicken’ moments of resilience and thriving in participants’ lives and to develop and sustain a positive therapeutic alliance. Participants are supported to develop a new relationship to depression (seeing depression as habits of mind they can learn to ‘live well alongside’ rather than as something that needs to be ‘cured’), and to rediscover or develop an identity beyond depression. The end phase of acute therapy builds a wellbeing plan to continue progress, and five booster sessions over the following year are offered to help sustain progress and trouble shoot any difficulties the participant may encounter. Figure 1 presents the logic model underpinning the intervention.

Preliminary evaluations of ADepT are encouraging, suggesting ADepT is an acceptable and clinically effective treatment for depression.^18 20^ A multiple randomised baseline case series treating 11 depressed individuals found ADepT was feasible and acceptable to participants and resulted in large effect size improvement in wellbeing and depression that were largely sustained across long-term follow-up.^20^ A pilot randomised controlled trial has also recently been conducted, where 82 depressed participants were randomised to receive either ADepT or 20 sessions of CBT.^18 24^ Again, ADepT was feasible to deliver and resulted in large effect size within-arm improvements in depression, wellbeing, anhedonia and other secondary outcomes that were sustained over time. While the pilot trial was not formally powered for between-group comparisons using frequentist statistics, inspection of between-group effect size confidence intervals using Bayesian approaches suggested that ADepT was very unlikely to be minimum clinically important difference (MCID) inferior to CBT (<1% likelihood on both depression and well-being outcomes) and showed potential to be MCID superior to CBT (>40% likelihood on depression and >60% likelihood on wellbeing outcomes). ADepT led to 80% of participants no longer meeting diagnostic criteria for depression according to clinical interviews at the 6-month primary outcome point, and 72% of participants met criteria for sustained remission across all of the 6-month, 12-month and 18-month assessments. Therefore, ADepT shows potential to enhance long-term depression outcomes relative to existing depression psychotherapies. Further, exploratory health economic analyses indicated that ADepT health-economically dominated CBT in this pilot trial (resulting in greater quality of life gains for comparable cost).

The quantitative outcomes are encouraging, but it is also important to evaluate complex interventions like ADepT from a broader qualitative perspective as part of a detailed process evaluation.^2225^,^28^ This can lead to iterative improvement and refinement of the intervention at early stages of research, to optimise the intervention before conducting definitive clinical trials. Qualitative user feedback can be used to test and update the logic model of the intervention (how an intervention leads to its effects) and enhance understanding of what conditions help or hinder this process (contextual modifying factors), referred to in the UK Medical Research Council process evaluation framework as programme theory.^25^ In particular, exploring participants’ views of the impact of treatment and how theses impacts were brought about can help identify mechanisms of change in treatment, what intervention elements modulate these mechanisms, and what factors influence the success of these intervention elements in real-world settings. These insights in turn can be used to further enhance treatment efficacy. Qualitative methods can also explore participant views on the feasibility and acceptability, defined as ‘a multifaceted construct that reflects the extent to which people delivering or receiving a healthcare intervention consider it to be appropriate, based on anticipated or experienced cognitive and emotional responses to the intervention’.^29^ Optimising acceptability will increase likely adherence to the intervention and implementation of the intervention both in subsequent definitive trials and in real-world implementation after trials.^25^

There has been only limited qualitative exploration of participants’ experience of ADepT to date. Thematic analysis of interviews with 11 participants who took part in the original case series on an early version of the ADepT protocol^20^ found that the wellbeing focus of treatment was acceptable, that treatment led to meaningful gains in wellbeing and reductions in depression, and that ADepT helped change their relationship to ongoing depression so that they were more able to be resilient when mood was low. Helpful elements of therapy were the positive therapeutic alliance that was established, clarifying values, enhancing engagement with simple everyday pleasures and optimising self-care. While therapy homework exercises were experienced as on balance helpful, for some participants the homework felt overwhelming. Given the small sample used in the case series,^20^ saturation was not reached. This qualitative analysis also did not explore perceived mechanisms of change in detail. Moreover, given the iterative nature of intervention development, it is important to further explore views of participants who underwent the updated protocol used in the subsequent pilot trial.^18 20^

To address this gap, the current study makes use of qualitative interviews with participants taking part in the trial to explore their experiences and views of the treatment.^24^ The following questions were explored:

1. To investigate acceptability of ADepT: what were participants’ views about the acceptability of the focus in ADepT on enhancing wellbeing versus reducing depression and which elements of the intervention did they find helpful or unhelpful?
2. To examine impacts of ADepT: what were participants’ views about perceived costs and benefits of undergoing treatment?
3. To provide insights into mechanisms of change within ADepT: how did participants account for any change that occurred during ADepT (and to what extent does this align with the logic model/programme theory of the intervention)?
4. To understand contextual modifying factors: what were barriers or facilitators to engaging with and benefiting from ADepT?

In addition to these research questions, the present study also examined any unexpected events or views of ADepT that arose from the data.^28^ Exploration of these questions will be used to propose further refinements to the treatment protocol to enhance the acceptability and potential efficacy of ADepT prior to proceeding to definitive trial.

## Methods

### Background trial design

This qualitative study was embedded within a two-arm, single-centre, open-label, mixed-methods, pilot randomised controlled trial of ADepT versus CBT for the treatment of anhedonic depression run in Devon, UK (ISRCTN85278228),^24^ which recruited participants between March 2017 and July 2018. 82 adults experiencing a current major depressive episode, according to the Structured Clinical Interview for Depression,^30^ scoring >10 on the Patient Health Questionnaire (PHQ-9)^31^ and with anhedonic features (scoring at least 2 on item one of the PHQ-9 measuring anhedonia [experiencing anhedonia for more than half the days in the past 2 weeks]), were recruited, primarily from local NHS Talking Therapy high-intensity waiting lists. Participants were randomised 1:1 (stratified by depression severity and use of antidepressant medication) to receive 20 sessions of CBT or ADepT, delivered in a university mood disorders specialist psychological therapy clinic by four experienced psychotherapists (either clinical psychologists or NHS Talking Therapies high-intensity trained therapists; two with prior experience of ADepT from the earlier case series and two who were new to the approach during the pilot trial). Participants were assessed at intake prior to randomisation and followed up at 6 months (primary outcome point), 12 months and 18 months after randomisation, with changes in depression severity (measured using the PHQ-9)^31^ and wellbeing (measured using the Warwick Edinburgh Mental Wellbeing Scale; WEMWBS)^32^ as co-primary outcomes.

### Qualitative process evaluation design

A mixed-method process evaluation was incorporated into the trial, including inviting participants in both arms to take part in a qualitative interview to explore their experiences of and views of treatment.^24^ All participants recruited into the trial were eligible to take part in these interviews and all initially consented to be contacted at a later point to schedule them (although we explained at the point of trial recruitment that only a subset of participants who consented would eventually be approached for the interviews). We here focus solely on interviews with participants in the ADepT arm.

Participants were approached to schedule the interview by the preferred method of contact they had given when initially recruited into the trial (either telephone, email, letter and/or video conferencing). If participants could not be contacted by this method, other methods they had given permission for were then also tried. Three attempts were made to book an interview with participants—if after this time no reply had been received, it was assumed they no longer wished to take part.

The interviews lasted between 25 and 70 min and were predominantly conducted face-to-face over a single meeting in the research clinic hosting the trial, although some participants instead requested interviews be conducted over the phone or via video conferencing. Interviews were conducted by research assistants or trial managers working on the trial (including coauthors EW and LW), with no one else being present beside the participants and researcher. All interviewers were female and had a background in mental health research and practice, including having completed at least an undergraduate degree in psychology or a related discipline (and a majority had already or were in the process of completing a research-related masters). Interviewers received training in qualitative interviewing from the trial principal investigator (BD), who in turn was mentored and supervised by an experienced qualitative methodologist. The interviewers had no prior relationship with participants before the trial was conducted, but had often engaged with participants prior to completing the interviews as part of broader trial procedures and data collection. Participants knew the reasons for doing the research from the study information sheet and having written, informed consent taken for the interview component of the study. Participants knew that the interviewers were part of the broader trial team and the interviews were conducted as part of this role. All interviewers had an interest in developing a research or clinical career in mental health and were aware of the aims and objectives of the ADepT intervention (see online supplemental material for a copy of the topic guide and notes on how questions and prompts address the objectives of the current study).

Interviews followed a topic guide to ensure broad experiences of treatment were covered, which was piloted prior to the study. Acceptability was investigated via questions around views on the well-being focus of ADepT and which elements of treatment were seen as helpful or unhelpful. Impacts were identified via a series of questions of immediate and long-term consequences of treatment. Contextual modifying factors were explored via questions about barriers and facilitators to engagement with ADepT. Given the centrality of positive therapist style to the ADepT logic model, a series of questions explored participants’ views on the positive, solution-focused and future-oriented therapist style of ADepT and how this impacted their relationship with the therapist (ie, formation of a positive emotional bond). There were no other explicit questions about participants’ views on mechanism of action of ADepT, but these themes spontaneously emerged in response to other questions. The distinction between what is an impact of treatment and what is a mechanism of treatment is not clear-cut. For example, initial change in interpersonal communication style could be seen as an early impact, which in turn could lead to subsequent improvement in mood (and hence is also a mechanism of change. Here, we use *impact* to refer to changes in the high-level objectives of ADepT to enhance well-being and functioning over the long time. We use *mechanism* to refer to any change that occurs during therapy that helps bring about the desired outcomes, which may or may not be a more a proximal impact in its own right (including changes in thinking, feeling, body and behaving). There were also questions exploring views on engagement with the research process, which are not the focus of the current study and so are not considered further here. Interviewers were also encouraged to explore any additional issues or themes that emerged in the interviews. The topic guide was continuously updated based on incoming data during interviews. At the end of the interviews, participants rated the acceptability of treatment (ranging from 1—‘not at all acceptable’ to 5—‘extremely acceptable’), satisfaction with treatment (ranging from 1—‘not at all satisfied’ to 5—‘extremely satisfied’) and the likelihood of recommending treatment to others (ranging from 1—‘extremely likely’ to 5—‘extremely unlikely’). The interviews were recorded on an encrypted digital recorder and then transcribed verbatim by a professional transcriber, apart from two that were transcribed by a member of the research team due to resource constraints. At this stage, the transcripts were anonymised (removing client or therapist name or other information that could identify people). Interviewers did not take field notes.

Minor changes were made to the preregistered study design.^24^ The original intention had been to use purposive sampling to recruit a representative subset of participants who did versus did not complete a minimum adequate dose of ADepT (at least 50% of the acute therapy dose; eight sessions), and to a select a combination of participants who did versus did not show reliable improvement on the primary trial depression and/or well-being outcomes, continuing to interview until data saturation was reached (target sample per arm of around 15 individuals). However, due to most participants engaging fully with and benefiting from ADepT (and those who did not dropping out of the trial entirely), these purposive sampling aims were impossible to achieve. In particular, of 41 participants randomised to ADepT in the trial, 40 initiated treatment and 36 had a planned discharge (35 of whom completed a minimum adequate dose of at least eight sessions [50% of acute treatment dose]). Of 34 ADepT participants completing 6-month assessment, 31 showed reliable improvement on the PHQ-9 and/or WEMWBS. Therefore, we moved to a convenience sampling approach, interviewing sufficient individuals until information saturation had been reached.

The preregistered intention had also been to interview participants between the 6-month and 12-month follow-up in the trial,^24^ but not all participants had completed acute treatment at 6-month follow-up and it was not always possible to schedule an interview within the following 6-month window due to logistical factors. The scheduling of the interviews was largely dictated by practical constraints (participant and research team availability). In particular, the interviews took place towards the end of the trial, when workload demands had reduced on the research team in terms of ongoing recruitment and outcome data collection, meaning they had more time to engage in interviewing participants. Therefore, those participants who had been recruited into the trial earlier tended to have a longer period between the end of treatment and the interview, compared with those recruited into the trial later.

Participants had their travel costs reimbursed and received a £10 honorarium for taking part in the qualitative interview. The conduct and reporting of this qualitative process analysis is informed by the Standards for Reporting Qualitative Research^33^ and the Consolidated criteria for Reporting Qualitative research.^34^

### Qualitative data analysis

Analysis was informed by the Framework Method,^35 36^ taking an integrative deductive and inductive approach to ensure that the research questions were addressed adequately, while there was also room to explore interviewee’s broader experiences of ADepT. A pragmatic stance was adopted, aiming to develop practical understanding of concrete, real-world issues that can inform action,^37^ while at the same time recognising that an objective reality cannot be comprehended perfectly due to the impossibility of eliminating any researcher’s subjectivity to the analytical process (a postpositivist viewpoint).^38^ The primary author and analyst of the current study, Kalliopi Demetriou (KD) did not conduct or transcribe the interviews. Following stages recommended in the Framework Method approach,^35 36^ the analyst first familiarised herself with the data (reading the first 10 interviews carefully and keeping a reflective memoir). The first three interviews were then open-coded, and individual mind maps were created to visualise the key themes in each of these interviews. One of these interviews was also read and open-coded by each of the supervisor BD and qualitative collaborator RW, with KD meeting separately with both to discuss each interview and compare and refine the coding schemes.^39^

An overall working analytical framework was then developed, consisting of a hierarchical structure of themes, subthemes and codes, to guide the coding of the rest of the interviews. This framework was partly informed by the research questions set and by a series of deductive influences, including the logic model underpinning the ADepT intervention and previous qualitative analyses of experiences with a related positive CBT intervention for depression.^20 40^ All interview transcripts were imported into the NVivo software,^41^ to assist in systematically applying the analytical framework to the remaining interviews (with the coding framework being iteratively updated as new themes arose). When all interviews had been coded, a framework matrix was generated to identify key themes, compare these between participants and map connections between themes. This analysis was submitted by KD for a Masters qualification. BD, KD and KM then condensed this thesis into a shorter form for the current manuscript and integrated a lived experience perspective into the findings (KM has received a course of ADepT for depression outside of this trial). To retain equipoise in the analysis and to maximise a critical analysis of ADepT at the point the current manuscript was drafted, interviews were revisited from participants who gave mixed, neutral or negative quantitative ratings of their experience of ADepT and from participants who did not show reliable improvement on either of the two primary outcomes. The analysis was updated to ensure the perspectives from these participants were fully incorporated (see online supplemental material for considerations of sources of potential analyst bias and for full details of how coding was conducted). Changes recommended by KM were to further emphasise the importance of building hope and of a ‘human’, ‘alongside’ therapist style.

### Patient and public involvement

The content and form of the ADepT intervention and the pilot trial evaluating it was co-designed using input from the public and patient involvement (PPI) lead on the project team (NR), members of the Lived Experience Group (LEG) at the Mood Disorders Centre, University of Exeter, and qualitative interviews with service users. PPI members were involved in all governance and delivery of the project, including NR meeting regularly with the principal investigator, attending ADepT project team meetings, being a coauthor on all trial publications, and contributing to the dissemination of findings. Another LEG member participated in the combined trial steering committee and data monitoring committee. With regard to the qualitative interview components of the trial, PPI members helped develop the topic guide and assessed the burden and possible harms of participation in the qualitative interviews in the present analyses. Coauthor KM has received ADepT (outside of the present trial) and helped check if the present analyses reflected service users experience of treatment.

## Results

Interviews were conducted between May 2018 and February 2020 and occurred between one and 25 months following the end of acute treatment. Of 41 participants recruited into the ADepT arm, five participants dropped out of the research before the interview phase, leaving a possible pool of 37 participants. Twenty participants were contacted, continued to consent to the interview and were interviewed (49% of individuals treated with ADepT in the trial). A further three participants were contacted, but no longer consented to take part in the interview component of the research. Two participants cited being too busy, while one mentioned that she would struggle with spoken English, at it was her second language. We were unable to contact eight participants. We did not attempt to contact the remaining six participants, as data saturation had been reached and the trial was coming to an end. The group-level demographic, clinical and treatment characteristics of participants are shown in table 1 (see online supplemental table S1, for individual-level clinical and treatment characteristics). The sample interviewed were a mixture of genders and ages and were predominantly of White British ethnicity. They presented with elevated levels of depression, anxiety and anhedonia and languishing levels of wellbeing prior to treatment. Ten participants were interviewed who had been treated by the two therapists with prior ADepT experience and 10 participants were interviewed who had been treated by the two therapists new to the approach in the trial. Reflecting strong engagement and promising clinical outcomes with ADepT in the broader trial sample, all but one participant interviewed had completed a minimum adequate dose of therapy, and all but three had shown reliable improvement in wellbeing and depression outcomes. Eighteen of 20 participants no longer met diagnostic criteria for depression on clinical interview (ie, were in remission) at the 6-month primary outcome. Nineteen participants had diagnostic data at each of the 6-month, 12-month and 18-month assessment, 13 of whom met remission criteria at all time points (ie, showed sustained remission). Only one participant (AD88) did not meet remission criteria at any assessment. Therefore, the sample consisted predominantly of individuals who showed sustained benefits from ADepT. Twelve participants were interviewed after completing the booster phase, six during the booster phase, and one participant did not engage with the booster phase of therapy.

**Table 1:** Clinical and demographic features of ADepT participants who participated in qualitative interviews

Variable	Mean (SD) or count (%)
Age	45.80 (13.65)
Gender	9/20 female (45%)
Ethnicity	18/20 white British (90%)
Intake (PHQ-9) depression severity	17.75 (3.52)
Intake (WEMWBS) wellbeing	31.01 (6.99)
Intake (GAD-7) anxiety	13.70 (3.66)
Intake (Snaith Hamiton Pleasure Scale; SHAPS) anhedonia	32.89 (6.12)
Taking antidepressant medication	11/20 (55%)
Previous psychological therapy	15/20 (75%)
Mean acute sessions attended (out of 20)	14.55 (1.61)
Mean booster sessions attended (out of 5)	3.75 (1.89)
Minimum adequate dose completed (eight or more sessions)	19/20 (95%)
PHQ-9 reliable improvement at 6 months (>5)	15/20 (75%)
WEMWBS reliable improvement at 6 months (>4)	16/19 (84%)*
PHQ-9 reliable deterioration at 6 months (<–5)	0/20 (0%)
WEMWBS reliable deterioration at 6 months (<–4)	2/19 (11%)*
PHQ-9 recovery at 6 months (<10)	15/20 (75%)
WEMWBS recovery at 6 months (>42)	12/20 (60%)
Acceptability ratings	7 extremely, 12 very, 1 moderately
Satisfaction ratings	7 extremely, 9 very, 4 moderately
Likelihood to recommend ratings	10 very likely, 10 likely

Note: cContinuous data are mean (one1 SD) values; categorical data are count (%) values.

* One participant had missing WEMWBS data at intake.

ADepTaugmented depression therapyGAD-7General Anxiety Disorder-7PHQ-9Patient Health QuestionnaireSHAPSSnaith Hamilton Pleasure ScaleWEMWBSWarwick Edinburgh Mental Wellbeing Scale

Quantitative ratings indicated that all participants interviewed found treatment at least moderately acceptable, were at least moderately satisfied with it, and would be very likely or likely to recommend ADepT to others. Quotes from the four participants who rated ADepT as only moderately acceptable (AD30, AD31, AD88, and AD91), so were less clearly positive about the approach, are suffixed with an asterisk. Quotes from the three participants who did not show reliable improvement on either of the depression or wellbeing primary outcomes (AD31, AD59 and AD88), and so less clearly gained benefit from treatment, are suffixed with a plus. There was no obvious clustering by therapist for participants who were only moderately satisfied or did not show reliable improvement.

Qualitative results are presented under four main headings: treatment acceptability; impact of therapy; mechanisms of change; and barriers and facilitators to engagement, reflecting our four primary research questions (see table 2 for an overview of themes).

**Table 2:** Summary of themes emerging from qualitative analysis

Area of exploration	Theme
Focus of therapy	1.1 Building resilience and thriving in the future, rather than taking away psychopathology
	1.2 Change in wellbeing and depression being intertwined
Helpful and unhelpful aspects	2.1 Positive therapeutic bond (+)
	2.2 Structure and flow scaffolding therapy (+)
	2.3. ADepT tools and techniques building and sustaining wellbeing (+)
	2.4 Booster sessions sustaining long-term recovery (+)
	2.5 Intensity of therapy (+/−)
	2.6 Therapy as too hard work and participants feeling they sometimes ‘failed’ (−)
Impacts of therapy	3.1 Reconnecting to wellbeing: pleasure, meaning and social connection (+)
	3.2 Increasing functioning in the face of low mood and life challenges (+)
	3.3 Finding hope (+)
	3.4 No change or change unrelated to ADepT (−)
Mechanisms of change	4.1 Attending to the positives (+)
	4.2 Starting on a values-based living path (+)
	4.3 Establishing a healthier lifestyle (+)
	4.4 Adopting a proactive life stance and taking control (+)
	4.5 Noticing old patterns and taking opposite action (+)
	4.6 Changing relationship to depression and rediscovering themselves (+)
	4.7 Cultivating self-care and self-compassion (+)
	4.8 Enhanced interpersonal effectiveness (+)
Barriers and facilitators	5.1 Readiness for, and openness to, change (+)
	5.2 A supportive network (+)
	5.3 Therapist flexibility (+)
	5.4 Life challenges getting in the way (−)

Note: +=helpful, −=unhelpful.

ADepTaugmented depression therapy

### Acceptability of ADepT

Acceptability of therapy was explored in terms of views on the wellbeing focus of ADepT and perceived helpful and unhelpful elements of treatment. Most participants reported that they recognised that the overall aim of ADepT was to build wellbeing in the present and the future, that this felt more acceptable to them than solely reducing depression, and that this wellbeing emphasis helped them step away from sometimes unhelpful rumination about the causes, meanings and consequences of depression (building resilience and thriving in the future rather than taking away psychopathology; theme 1.1):

It wasn’t or it did not appear to me to be a subtractive process, it was an added upon, I’m not trying to take away a problem I have, I was trying to add something or return something that had gone missing. So yeah, it was focussing on how can we make this better, not how can we stop this being bad. (AD66)

It’s more about where we can go from here, rather than going over what was in the past, which I think was good for me. I’ve done enough ruminating, it’s good to think about how you can change things going forward. (AD49)

Nevertheless, enhancing wellbeing and reducing depression were seen as two sides of the same coin, so building wellbeing then also reduced depression symptoms and vice versa (change in wellbeing and depression being intertwined; theme 1.2):

It seemed to be a lot of combination. I mean, I don’t think you can do the one, sort of fighting depression, without at the same time building the wellbeing. (AD54)

Further suggesting ADepT was acceptable, participants commented on a variety of elements of treatment they found helpful, including therapeutic bond, therapy structuring and flow, ADepT specific tools, and booster sessions. A helpful element of therapy that nearly all participants talked about was the positive bond they built with their therapist (positive therapeutic bond; theme 2.1), fostered by the therapists’ interpersonal style. Participants mentioned a range of positive qualities that they saw in their therapists, including ‘friendly’, ‘warm’, ‘completely non-judgemental’, ‘open’, ‘respectful’, ‘honest’ and ‘invested’. Core to this experience was participants being treated as a ‘human’ by another ‘human’ first and foremost (rather than as a ‘bunch of symptoms’), with sessions being tailored to their needs and feeling ‘very personal’. The open therapist style (with judicious use of therapist self-disclosure and humour), and a willingness to listen to and act on participant feedback felt authentic, built common ground with participants, and helped them to feel the therapist was walking alongside them (a collaborative journey).

Rather than this is the stages you go through, it’s a case of, right, how can we help you, what do you feel comfortable with, what don’t you feel comfortable with. (AD18)

Felt like …. [therapist] was talking to me individually and he tailor-made the solutions to fit my problems specifically. (AD69)

And we (therapist and client) could laugh about certain things, which was very, very good and this was helpful. So it wasn’t just someone sitting there and trying to, it was more the part of life experience we’ve got together, which was extremely helpful. (AD51)

Participants spoke favourably about the length, duration, structure and spacing of sessions within ADepT (structure and flow scaffolding therapy; theme 2.2). Participants liked the sense of logical flow and continuity of sessions, including homework tasks building logically on current session content:

The scaffolding approach of it (…) it felt like it was a supported journey, that each week was building on what I’d been doing the week before, and it felt like there was an evolving process. So rather than it being a repetition of the same thing, it felt like there was a clear demonstration for me that I was moving forward. (AD22)

ADepT-specific tools and techniques were also experienced as helpful including mood diaries, simple pleasures exercises, diary of thriving and resilience, the mapping tool and the values dartboard (ADepT tools and techniques building and sustaining wellbeing; theme 2.3). Using these tools in session and homework helped consolidate learning, facilitated change, and supported ongoing progress after therapy:

Without writing things down, it wouldn’t have helped me, I like to put things down in writing because it kind of reinforces what I’m doing, if that makes sense. (AD49)

And I felt like that (dartboard) was really, really helpful, because it helped me visualise like where am I putting more time and where I could drop …. like cut time out of where I needed to, if that makes sense. (AD59+)

So every morning they (handouts) are still quite good, I’ve still got them on my desk for times when I’m starting to feel low. (AD18)

Flexible scheduling of booster sessions after acute treatment was experienced as helpful for promoting long-term recovery. These sessions helped smooth out the end of therapy, allowed space for participants to try things on their own and come back to reflect on these and to get support when needed on tackling new situations:

And then the sort of follow-ups to help you go through some, if you've got some challenging things coming up, then you’ve got that support afterwards so that you can tackle the next big thing, whereas if you didn’t have that follow-up appointment it might just knock you right back, having had a big challenge and failed or something. (AD49)

There was a general recognition that change during ADepT was hard work (intensity of therapy; theme 2.5), which was both a good and bad thing. At times, engaging with ADepT was intense, difficult and draining, requiring participants to push beyond their comfort zone to make significant life changes. At times, some participants felt overwhelmed with paperwork and home practice and struggled to fit this into already busy lives. Nevertheless, most reported this effort was ultimately useful:

One hour was too long for me, I was very tired and drained. (AD31*+)

I mean, it’s quite hard really, because I think I ended up with a long list, even the last session I had with (therapist) I ended up with a long list of things—she said, try to do this, try to do that—and you sort of think, yeah, but on a daily basis it’s quite hard work. (AD74)

The homeworks were a pain in the neck but useful. (AD66)

The intensity of ADepT was off putting for a minority of participants, leading to self-criticism if they did not meet all of their goals, impacting adversely on their alliance with the therapist and making some participants consider disengagement from treatment (therapy as too hard work and participants feeling they sometimes ‘failed’; theme 2.6):

Some of the days when I’ve come in here and he’s [therapist] gone through some of the paperwork and the targets and the goals and that, has felt a bit pushy to me. (AD30*)

I’m not a very good organised person, so making a plan and didn’t do it, it was always bigger stress for me …… I always have bad feelings when I didn’t do something from my list. (AD31*+)

I suppose it makes me more aware of the responsibility, the challenge, that I’ve got personally to manage it [depression]. It’s a double-sided coin. I’m more aware that, by doing the right things and thinking clearly enough and being consistent enough, I can have an effect on my mood and my life more generally in a positive way, so that’s good. The flip side of that is, I suppose, that if I’m having a bad week in terms of mood … I’m more aware that that’s my fault, so I can blame myself for it more. (AD88*)

### The impacts of ADepT

ADepT led to perceived improvements in wellbeing and functioning alongside symptom relief for most participants. A majority of participants reported increases in the capacity to seek out and enjoy rewarding experiences in life, to reconnect to what is important and meaningful to them, to strengthen a sense of being socially connected to (and valued by) others and/or feeling more engaged in life (reconnecting to wellbeing: pleasure, meaning and social connection; theme 3.1):

And what we [client and therapist] did was to, within the moment is to really stop and think about why you enjoy it [leisure activities]. And then that was quite a great thing because then that gave me even more pleasure, it kind of reminded me why I do these things and why I like it. (AD48)

I still am very aware to stay in contact with people and talk to people, like friends and family. And I’m more aware to keep those contacts going than I probably did before the therapy. (AD54)

I’ve got much more involved…. I’ve picked up on some things that I’m interested in and try to put some energy in those. (AD54)

For some, there were significant positive transformation across multiple areas of life that had been noticed by other people in their network:

For me, I think it’s changed my life basically. Not saying that easily, but it’s just changed the way that I look at things and approach things now. (AD49)

And a colleague I worked with, I only spoke to him yesterday and he said to me ‘this time last year’ he said, ‘the difference the year makes, the change in you is phenomenal and your life has completely turned around’. (AD48)

A number of participants reflected that while they still experienced periods of low mood and faced life challenges, they felt more able to cope with these difficulties and to continue to function due to the resilience skills ADepT had given them (increasing functioning in the face of low mood and life challenges; theme 3.2). There was an increasing awareness that these moments of low mood were only part of the picture:

I’m still having very low moments, but through this therapy I’m going back to the techniques me and [therapist’s name] have used, and I haven’t been off sick from work and I haven’t stayed in bed. So all I can say is that it’s worked. (AD32)

Treatment also helped participants reconnect to a sense of hope during treatment, recognising the possibility of a positive life ahead (finding hope; theme 3.3):

Seeing that there would be life ahead of me. (AD49)

Particularly helpful in this regard was the ‘living well alongside depression’ emphasis in treatment, with participants discovering they could experience moments of wellbeing worth living for even in the midst of significant depressive symptoms. Furthermore, participants described therapists as having a key role ‘holding the hope’ at times their mood was particularly low.

However, a minority of participants did not attribute the positive changes they experienced as related to ADepT and some reported very little change at all (no change or change unrelated to ADepT: theme 3.4):

I think the medication really helped. I think because I’d done a lot of counselling previously, I kind of knew what I had to do. But because my depression was so bad, that the anti-depressants kind of took a weight off. (AD91*)

The only thing I’ve really gained from it is the fact that it’s got me out the house and it’s been somewhere to come, somewhere different, somebody to chat to. The therapy side of it, it didn’t really sort of do anything for me. (AD30*)

### Mechanisms of change in ADepT

A number of mechanisms of change that contributed to beneficial outcomes from ADepT were described in the interviews, including a positive attentional focus, finding hope, clarifying values, building healthy routines, becoming more proactive, acting opposite to old habits, changing relationship to depression, cultivating kindness to the self, and improving interpersonal skills. Participants commented on how ADepT helped them to acknowledge, attend to and engage with positive external events and personal achievements in their lives (‘glimmers of light’), at both an intellectual and experiential level (attending to the positives; theme 4.1). Often these positive moments had been overlooked or minimised before therapy:

I practised putting a positive spin on your life so you are saying ‘… but hang on what is the positive aspect of this?’. So you might say ‘oh I have achieved nothing today’ but actually, I set out to do 5 things and I have done 2 of them so I am happy with those 2 and I got halfway into the third one and that’s a good thing. (AD82)

Many participants commented that ADepT helped them explore and clarify their values in life to reconnect to what was important to them, which in turn helped them to set and prioritise between different goals across, and to find a balance between, life domains (starting on a values-based living path; theme 4.2):

I think it’s just finding my own values in life. I never stopped to think about it, because I was always too busy helping other people and just running on a treadmill, keeping life going. (AD49)

They’ve (therapy) made me put it into perspective of what’s important. Again, coming back to that whole dartboard thing, it made me take into consideration the different aspects and what I needed to put more effort into and what I needed to be focusing on, if that makes sense. (AD59)

Participants also reported that therapy helped them establish healthier behavioural patterns routines, and reintroduce structure to their lives (establishing a healthier lifestyle;theme 4.3):

To me it was all pointed on building structure to your life to help you move forward. (AD30*)

I can see the value and I do think I got some benefit from the focus on trying to map out what might be more kind of constructive activities and (…) things that might lead me to a better state of mind (…) (AD88*+)

Working towards values-consistent goals in a structured way helped participants build a sense of agency and take back control of their lives (adopting a proactive life stance and taking control;theme 4.4). Breaking goals down into manageable steps and starting to progress with these steps helped participants build a sense of confidence in themselves and therapy and further motivated them to engage with treatment. Therapists fostered this process by noticing positive changes, encouraging a solution-focused rather than ruminative outlook, encouraging participants to recruit broader social support, introducing accountability regarding reviewing completion of homework tasks that had previously been set, and gradually handing increasing control of therapy over to participants:

I feel like I’ve learnt from this one (ADepT), I’ve walked away and gone, okay, I can try and deal with everything on my own now. (AD48)

And it basically made me believe in myself. And I’ve been able to say that I’m proud of myself for the first time in, oh my God, seven or eight years, because of how hard I work, the times when I want to phone in sick and I don’t. (AD32)

So it made it achievable, breaking down the tasks into smaller little goals, even if it was only like get up in time to do something, a simple goal like that, but that sort of gets you out of bed and gets you motivated. (AD49)

Because like [therapist’s name] used to say to me, which is really good, is that I’m your driving instructor, which I thought was a great metaphor—so I’m going to teach you this, but then you’re going to go off on your own. Because I used to rely on [therapist’s name] a hell of a lot. (AD32)

Key to building this sense of self-efficacy was developing skills to spot early and skilfully respond to depressogenic patterns and to choose to respond in a different way (noticing old patterns and taking opposite action; theme 4.5). For example, where participants noticed the urge to avoid, they would instead approach a situation:

I think it’s the ability to spot the signs, and then once you can spot the signs then you can take a step to prevent it. (AD69)

Participants also commented that ADepT helped them change their relationship to depression, seeing depression as just one part of the self, or for some a separate entity from the self, rather than something that defines them (changing relationship to depression and rediscovering themselves; theme 4.6). Participants talked about gaining a better understanding of depression and its chronic nature, but also of the fact that they can make progress in their life despite the depression (‘living well alongside’):

I used to think of my depression as something that’s always defined me and has always been a part of me, because I’ve had depression since I was quite young […] Whereas actually there’s a lot of things that define me other than my depression, and…. this therapy helped highlight that actually, I’m so much more than that. (AD79)

I suppose it’s coming to an understanding of what it is and how to live with it [depression], rather than there is no, or didn’t seem to be any, cure for it’ It’s more managing it and understanding that it’s okay sometimes to feel bad and not let that become a vicious spiral. (AD69)

Many participants reported that ADepT helped them adopt a more compassionate attitude towards themselves (cultivating self-care and self-compassion; theme 4.7). This was expressed at the cognitive level (recognising their self-critical voice and replacing it with a kinder voice), and at the behavioural level (engaging in more self-care activities):

I’m not as judgemental on myself. (AD18)

And I think one of the big things for me was about self-care, returning to a position of self-care and putting myself as an important part of my life, I suppose. So things like eating well, reducing alcohol intake, increasing exercise, being kind to myself. (AD22)

Many participants reported changes in their relationship to depression and self-compassion in turn changed how they communicated with others, becoming more willing to share their difficulties and communicate their needs (enhanced interpersonal effectiveness; theme 4.8). This in turn left them feeling less lonely, estranged, alienated and ashamed, as they were often met with understanding, validation and kindness from others, including learning others also had suffered from low mood:

….being able to actually open up and tell people that I do have a problem. And sharing with other people. Because, the more I’ve shared with people, the more I’ve learnt that people are struggling themselves. (AD64)

It [ADepT] has helped change the way I look at things, makes me more assertive, which I think helps my well-being as well. Because before I was used as a bit of a floor mop, just to circumvent the office problems. (AD49)

### Barriers and facilitators to engagement with ADepT

A variety of facilitators and barriers to engagement with ADepT (contextual modifying factors) were identified. Feeling motivated to change at the start of ADepT was perceived as being key to being able to benefit from it (readiness for, and openness to, change; theme 5.1). Some participants commented that, having tried a series of symptom-focused approaches previously with mixed outcomes, the wellbeing and ‘living well alongside’ emphasis of ADepT felt something different to try. Participants also commented that coming into therapy with a sense that depression was their ‘problem to own and manage’ meant they were ready to run with the ADepT framework:

That is the biggest factor in all of it, is (1) only you can fix it, and (2) you’ve got to want to fix it. (AD32)

I think it was just the fact that I was sick and tired of being down in the dumps 90% of the time, and I just wanted to make myself feel better in my head. (AD59+)

Some participants acknowledged ambivalent motivation to engage with tasks linked to therapy in early stages, but as treatment delivered gains, this ambivalence reduced:

…it was more I didn’t want to do any of it, but I was willing to give it a go, I thought what’s the worst that could happen, I can try and it wouldn’t work and then I could tell (therapist) he was an idiot. But I was never able to do that because it kind of worked. And even though I didn’t particularly want to do it, I didn’t particularly enjoy doing it, it was I saw the benefits of doing it afterwards. (AD69+)

While participants needed to want to ‘own’ the work, at the same time they benefited from being open to work collaboratively with a therapist and take constructive advice. Where this willingness to collaborate was not established, it reduced perceived engagement with and benefit from ADepT:

I don’t tend to sort of listen to what people … well, I do listen, but I sort of tend to still do what I want to do, when I want to do it, how I want to do it. (AD30*)

Having supportive individuals around them to reinforce positive change and to consolidate learning also helped individuals engage with ADepT (a supportive network; theme 5.2):

My employer in my other, in one of my jobs, has been really understanding and you know hasn’t put any pressure on me at all and is still checking in now with me, you know, how are you, you know, how’s it going, how are you doing, you know, he’s still checking with me so that’s been good, that helps. (AD93)

Having flexibility around session structuring and delivery format also supported engagement (therapist flexibility; theme 5.3):

Staff were really helpful, like if I was unable to attend a meeting I could email and then obviously that would get sorted out as far as like attendance-wise (…) But it was really nice to have that sort of, again, flexibility. (AD59+)

External life challenges (adverse life events, poor physical health and too many other things going on) at times were a barrier to engaging with ADepT (life challenges getting in the way; theme 5.4):

Unfortunately, a lot of my problems are family issues, so it was difficult to sort of really sort those out. So that’s quite a difficult one to deal with. (AD74+)

But all the stuff that we’'ve (participant and therapist) gone through, and we’ve done, I mean to me it’s pretty, life structures, it’s putting structure back into your life, isn’t it (…) I just can’t do it. Things like getting up early in the morning and having meals at set times and things like that, it’s very difficult for me to do because of having the fibro(myalgia) the way I’ve got it. (AD30*)

## Discussion

The current qualitative study explored participant perceptions of the acceptability, impacts, mechanisms of action and contextual modifying factors of ADepT, using these insights to further refine the treatment protocol and to help inform the decision about whether or not to proceed to definitive trial. Twenty participants were interviewed, exceeding the target within-arm sample size estimated in the trial protocol paper and estimates from meta-analyses of a number of interviews required to reach saturation.^24 42^ Reflecting the fact that a majority of participants engaged well with and benefited from ADepT in the trial, the study purposive sampling aims were not fully achieved (including only one participant not completing a minimum adequate dose and only three participants not showing reliable improvement on either depression or wellbeing outcomes). Participants were a mixture of ages and genders and most were of White British ethnicity, reflecting the sample treated in the trial and the demographics of the Devon area of the UK that the trial recruited from.

Regarding acceptability aims, participants quantitatively rated therapy as acceptable (16/20 fully satisfied and 4/20 moderately satisfied). Further suggesting that ADepT was acceptable, interviews revealed the wellbeing focus of ADepT was valued by participants and that they had found a variety of treatment elements helpful, including the positive therapist style, the structure of therapy, specific ADepT tools and techniques and the availability of booster sessions. However, for a minority of participants, ADepT was experienced as extremely hard work at times of low mood, triggering judgements of ‘failing’ in therapy. These findings accord with previous quantitative findings from the earlier ADepT case series and pilot trial, which found that participants were willing to engage with ADepT, there were low rates of drop out and a vast majority completed the full dose of acute therapy and engaged with optional booster sessions.^18 20^ Findings also align with preliminary qualitative exploration of experiences in ADepT in the case series, where participants described valuing the well-being focus and that many aspects of therapy were helpful, but that treatment was at times hard work. The importance of therapy structure in turning insights in session into lasting behavioural change accords with similar themes in participants’ accounts of benefits from CBT for depression.^43^

Regarding impact aims, a majority of participants described diverse positive impacts of ADepT including: enhancing key pleasure, meaning and social connection domains of wellbeing; strengthening functioning in key life domains (even alongside periods of low mood or life challenges); building a sense of hope about the future, with improvements ranging from modest to transformational. However, a minority of participants reported little improvement or did not attribute their improvement directly to ADepT. These outcomes echo the quantitative results in the earlier case series and pilot trial, with large effect-size increases in wellbeing and positive affect, and large effect-size decreases in depression and negative affect. Additionally, a majority of participants showed reliable improvement in these outcomes.^18 20^ Findings also align with preliminary qualitative findings from the case series,^20^ which emphasised a variety of wellbeing gains. That participants consistently commented on and valued wellbeing and functioning outcomes is consistent with participants’ lived experience accounts of depression, which emphasise a range of wellbeing-related deficits as central to the phenomenology of depression: an inability to experience positive emotions; feeling numb and empty; losing the capacity to act on the world; losing a sense of purpose and existential hope; a stagnation of the present and a sense of impossibility about a positive future.^10^ The importance of wellbeing to participants is also supportive of a greater emphasis on positive mental health outcomes emerging in the positive psychology and recovery literatures (eg, themes of connectedness, hope and optimism, identity, meaning and empowerment as key elements of recovery and as valued outcomes in depression).^13 44 45^ It is reassuring that participant accounts of change during ADepT demonstrate some degree of change in all of these elements. These themes are similar to those emerging from qualitative interview studies of related therapy approaches, including adolescents’ experience of Behavioural Activation^46^ and adult experiences of positive CBT,^40^ suggesting they are not unique to ADepT and also apply to other positively oriented treatments.

Regarding mechanism aims, participant perceptions of the impact and mechanisms of action of therapy fitted well with the ADepT logic model (figure 1). Participants described the benefits of a positive therapist bond (theme 2.1) and how ADepT helped them attend more to positives in a solution-focused fashion (theme 4.1), aligning with the emphasis in the logic model of building and sustaining a positive relationship with the participant to cultivate a solution-focused outlook (box 1 in figure 1). Participants reported that clarifying values and working towards values-consistent goals was beneficial, consistent with the emphasis on values exploration and behavioural activation towards values-consistent goals in the logic model (box 2 and box 3 in figure 1), and supporting the emphasis on values work in ADepT. Participants described having enhanced capacity to both maximise the impact of life opportunities to build wellbeing and positive emotions, and to minimise the impact of life challenges on depression and negative emotions (theme 3.1 and 3.2). This aligns with the logic model’s emphasis on building both resilience and thriving (box 5a and box 5b in figure 1) and also with ADepT’s theoretical emphasis on both downregulating the negative valence system and upregulating the positive valence system.^18 47^ Participants described how ADepT gave them skills to notice and respond differently to unhelpful depressogenic habits (theme 4.6), concordant with the emphasis on opposite action in the logic model (box 4 in figure 1). Participants commented on having a new relationship with depression (theme 4.7) and feeling they have more proactive control of life (theme 4.5), which accords with the emphasis on changing relationship to depression and enhanced self-efficacy to manage it in the logic model (box 6 in figure 1). Participants described benefiting from booster sessions to remain well and continuing their progress to values-consistent goals. This also aligns with the logic model’s emphasis on well-being planning for the future (box 6, figure 1).

Regarding contextual modifying factor aims, key facilitators to engagement with ADepT included readiness and openness to change when starting therapy, having a supportive social network and therapist flexibility around scheduling. Potential barriers to engagement were having significant life challenges (too much going on, poor physical health and/or major negative life events), particularly when therapy tools were not perceived as being able to help modulate or influence these external factors. These are similar to barrier and facilitator themes that emerged in qualitative studies of Behavioural Activation and CBT for depression.^48^ While these issues are not unique to ADepT, they illustrate the importance of therapists acknowledging and working with the ‘real-world context’ their clients are operating in to maximise participants’ engagement with and benefit from treatment.^22 25^

The present findings suggest a number of minor modifications that could be made to the ADepT logic model and intervention delivery to further enhance the intervention. Most importantly, some modifications are needed to ensure that therapists remain in participants’ ‘zone of proximal development’ for their current mood state, including adjusting current action steps and goals as mood state fluctuates within and between sessions. The ADepT rationale presented to participants could be explicit about the need to modulate expectations and to pace levels of activation as a function of current mental state. Furthermore, therapists need to more explicitly monitor for and respond to self-criticism being triggered when progress is not at participants’ desired pace. Suggesting areas for logic model refinement, participants also identified as key mechanisms of change enhancing interpersonal skills (assertiveness and help seeking) and building self-care and self-compassion, both of which are relatively implicit in the current logic model. The importance of enhancing hope as an outcome of treatment also suggests hope could be included more explicitly as an outcome target in the ADepT logic model. Treatment engagement could be enhanced by integrating more explicit ‘readiness assessments’ as used in Dialectical Behaviour Therapy at the start of treatment (and, if required, engaging in some precommitment work to build motivation, recruiting external support and problem-solving external life challenges to scaffold treatment engagement).^49^

Other suggestions for protocol refinement that emerged included simplifying tools used in therapy; being more explicit about the intended function of each session at the start to avoid drift and setting home practice expectations more clearly. A number of participants said they would have benefited from ongoing support after booster treatment had finished, for example, through peer-led drop-in support groups and access to a digital resource hub.

There are various study limitations to hold in mind. First, the participants in the current study represent a specific subpopulation of people with depression who self-selected and were screened to participate in the parent feasibility trial of ADepT (therefore were willing from the outset to engage in a novel wellbeing-oriented psychotherapy), and different views may emerge from routine care samples. Second, for pragmatic reasons, the timing of interviews varied considerably between participants, and for some, the interviews were delayed by up to 25 months following the end of the acute treatment phase. It is conceivable that participants’ views may have been modulated by whether or not they were still in the booster phase and that their memory of the intervention might have been limited for those interviewed a long time after treatment was completed. However, there was no discernibly different pattern of responses given as a function of treatment stage and only one participant commented that they struggled to remember their experiences with therapy. Third, participant levels of wellbeing and depression were not indexed at the precise point they completed the interview. This means we cannot rule out that participants’ views on treatment may have been influenced by their current mood state when interviewed. However, a majority of participants reported sustained remission on depression diagnostic interviews across all follow-up assessments, suggesting some homogeneity in disease status in those interviewed. Fourth, we did not include any formal participant member checking with those interviewed to ensure the themes that emerged were accurate and resonated with the experiences of those interviewed.^50^ However, coauthor KM has lived experience of depression and has undergone ADepT (outside of this trial). The analyses reported here broadly resonated with her lived experience. Fifth, existing work evaluating ADepT has been conducted with participants who received ADepT in a university research clinic setting. It is important to consider if these positive findings will also emerge in routine care settings. A recent pilot implementation study showed that therapists in routine NHS Talking Therapy settings could be trained to deliver ADepT with fidelity and competence, that this led to positive treatment outcomes with their clients and that therapists felt the model was effective and a good fit for NHS Talking Therapies settings.^51^ This provides preliminary evidence that effects are likely to hold in ‘real-world’ settings. Finally, the findings are at risk of allegiance bias, as the senior author (BD) on this paper is the developer of ADepT, the principal investigator on the ADepT pilot trial and served as a therapist and supervisor in the trial. To reduce potential bias, the primary analysis was done by KD, with no prior allegiance to ADepT. Steps were also taken to pay particular attention to themes emerging from less favourable interviews.

Overall, the present findings suggest that the wellbeing and functional recovery focus of ADepT is acceptable to participants, that most participants experienced meaningful gains during therapy and that participants’ explanations of how this positive change came about largely concurred with the logic model of the intervention. A number of suggestions emerged for further refinement of the intervention’s logic model and protocol. These findings further support ADepT as an emerging intervention option for anhedonic depression and warrant continuation to a definitive trial comparing (a slightly modified version of) ADepT with CBT in terms of clinical effectiveness and cost-effectiveness. More broadly, the findings highlight the value of placing an explicit focus on enhancing broader wellbeing and functioning in treatment for depression, rather than a narrow focus on symptom relief.

## supplementary material

10.1136/bmjopen-2024-088726 online supplemental file 1

## Data Availability

No data are available.
