# Supplementary material for: A qualitative study exploring depressed participants’ experiences of receiving Augmented Depression Therapy (ADepT)
Source: BMJ Open. 2025 Feb 5;15(2):e088726. doi: 10.1136/bmjopen-2024-088726 (PMC11800203; doi:10.1136/bmjopen-2024-088726)
Supplement: online supplemental file 1 [file bmjopen-15-2-s001.docx]

Supporting Online Materials for Demetriou et al ADepT qualitative paper

SOM Table S1: Participant clinical characteristics

| ID | Previous Therapy | Acute Sessions Attended | Min Dose | Booster Sessions Attended | Satisfied | Time since acute end (m) | Booster Phase | PHQ-9 Improver | WEMWBS Improver | SCID Remission 6m | SCID Remission All |
| --- | --- | --- | --- | --- | --- | --- | --- | --- | --- | --- | --- |
| AD18 | No | 15 | Yes | 4 | Yes | 25 | Post | Yes | Yes | Yes | - |
| AD19 | Yes (BC, CBT) | 15 | Yes | 3 | Yes | 18 | Post | Yes | Yes | Yes | Yes |
| AD22 | Yes (CBT, CT) | 15 | Yes | 5 | Yes | 9 | During | Yes | Yes | Yes | Yes |
| AD30 | No | 15 | Yes | 6 | No | 13 | Post | Yes | Yes | Yes | Yes |
| AD31 | No | 15 | Yes | 1 | No | 21 | Post | No | No | No | No |
| AD32 | Yes (IPT) | 15 | Yes | 5 | Yes | 5 | During | Yes | Yes | Yes | No |
| AD48 | Yes (CBT) | 15 | Yes | 1 | Yes | 0 | During | Yes | Yes | Yes | Yes |
| AD49 | Yes (CBT) | 15 | Yes | 5 | Yes | 15 | Post | Yes | Yes | Yes | Yes |
| AD51 | Yes (C) | 15 | Yes | 5 | Yes | 2 | During | Yes | Yes | Yes | No |
| AD54 | No | 15 | Yes | 5 | Yes | 18 | Post | Yes | Yes | Yes | No |
| AD59 | No | 15 | Yes | 3 | Yes | 13 | Post | No | - | Yes | Yes |
| AD64 | Yes (CBT) | 15 | Yes | 5 | Yes | 16 | Post | Yes | Yes | Yes | Yes |
| AD66 | Yes (CBT) | 15 | Yes | 5 | Yes | 17 | Post | Yes | Yes | Yes | Yes |
| AD69 | Yes (unspecified) | 13 | Yes | 1 | Yes | 7 | Post | No | Yes | Yes | Yes |
| AD74 | Yes (CBT) | 15 | Yes | 4 | Yes | 1 | During | No | Yes | Yes | No |
| AD79 | Yes (C, CBT) | 15 | Yes | 4 | Yes | 13 | Post | Yes | No | Yes | Yes |
| AD82 | Yes (CBT, MBCT) | 15 | Yes | 5 | Yes | 16 | Post | Yes | Yes | Yes | Yes |
| AD88 | Yes (CBT, MBCT) | 15 | Yes | 4 | No | 3 | During | No | No | No | No |
| AD91 | Yes (C) | 8 | No | 0 | No | 14 | - | Yes | Yes | Yes | Yes |
| AD93 | Yes (CBT, MBCT) | 15 | Yes | 5 | Yes | 12 | Post | Yes | Yes | Yes | Yes |

*Note.* ID = participant identifier. For treatment, C = Counselling; CBT= Cognitive Behavioural Therapy (for most participants this was low-intensity, short CBT); BC= Bereavement Counselling; CT=Couples Therapy; IPT=Interpersonal Therapy; MBCT =Mindfulness Based Cognitive Therapy. Minimum dose = attended at least eight sessions of ADepT. Satisfied = very or extremely satisfied. Booster Phase = interview conducted during or after booster phase (AD91 did not engage with the booster phase). PHQ-9 Improver= had at least a 6-point improvement on the PHQ-9 between baseline and 6-months follow-up; WEMWBS Improver = had at least a 3-point improvement on the WEMWBS between baseline and 6-month follow-up (*=showed at least a 3-point deterioration); AD59 did not have pre and post WEMWBS data, so improver status could not be calculated. SCID 6m = remitted at 6m; SCID all = remitted at 6m, 12m and 18m; AD18 did not complete 18m SCID interview, so sustained remission could not be calculated. Age, gender and ethnicity individual level data not provided to avoid patient identification.

Interview topic guide

*[notes in italics added at point of submission of this article to link topics back to research questions explored]*

1. Researcher to introduce themselves and explain the purpose of the interview.
2. Ask for consent to record- explain that this is so interviewer can give full attention without having to try and write it all down, and that recording will be transcribed and then analysed. “*We may quote your exact words when writing up the research but nobody will be able to identify you from them*…”
3. Participant to read and sign consent form

Opening question (an ice-breaker):

How did you find out about the research project?

Topic 1: Experience of Treatment

*[note: primarily to explore clients views on acceptability of treatment, often also spontaneously leading to clients talking about impacts and mechanisms of treatment]*

You have just received ADepT/CBT therapy. Could you tell me in your own words how you found this?

Probes (if required):

- Was it what you were expecting?
- Have you had therapy for depression before and, if so, how did it compare?
- Do you think this therapy focused more on reducing depression or building wellbeing?
- Did the therapy surprise you in any way?
- What was the best thing about it?
- What was the worst thing about it?
- Do you think there are any changes we could make that would improve your experience of the therapy?
- What did you think of the handouts and home exercises?
- Has therapy changed the way you think about your depression?

Topic 2: Therapeutic alliance

*[note: as a major element of ADepT is using a solution-focused style and adopting a positive interpersonal process, we wished to explore in detail clients view on the alliance they formed with the therapist, potentially informing understanding of acceptability and mechanisms of treatment]*

How did you get on with your therapist during ADepT/CBT therapy?

Probes (if required):

- How well do you feel your therapist related to you?
- Did you feel s/he understood you and wanted to help you?
- Did you feel that your therapist was actively involved in your treatment?
- Did you trust in your therapist?

Topic 3: Barriers to treatment

*[note: primarily to explore contextual modifying factors that helped or hindered treatment engagement]*

We are interested in things that help people fully engage with ADepT/CBT or prevent them from doing so. Please tell me what sort of things influenced how many sessions you attended and the amount of homework you completed during ADepT/CBT?

Probes (if required):

- If you dropped out of therapy before completing treatment, why was this?
- If you missed a session and then came back into treatment, what helped you to do this?
- Was the timing of sessions an issue for you at all?
- Was travel an issue for you at all?
- Were the views of friends and family an issue for you at all?
- Were other demands on your time an issue for you at all?
- Was your relationship with your therapist an issue for you at all?

Topic 4: Life after therapy

*[note: primarily to explore longer term impacts of treatment, given primary aim of ADepT is sustained reduction of symptoms, enhancing wellbeing and functioning, and reducing risk of relapse. Also often led to spontaneous exploration of mechanisms in action]*

What are your views about likely impact of CBT/ADepT in the longer term?

Probes (if required)

- Preventing relapse?
- Continuing to build wellbeing?
  - Vocation/Leisure/Self-Care/Relationships

Topic 5: Experience of research

*[note: to help assess acceptability of trial research procedures, not focused on in the current manuscript]*

How did you find taking part in the research study?

Probes (if required):

- What was your motivation to take part in a research study?
- How did you find completing weekly questionnaire measures?
- How did you find completing the questionnaire pack before and after treatment?
- How did you find completing the interviews before and after treatment?
- How did you find completing the experimental tasks before and after treatment?
- How did you find the volume of measures we asked you to complete?
- How did you find your contact with the research team (other than your therapist)?

Quantitative ratings:

[These questions and the rating scale were shown to participants on a laminated card. After making each ratings, participants were asked to explain why they answered that way with a probe question]

Overall, how acceptable was ADepT/CBT to you? In other words, did you think that the treatment approach and activities made sense and were reasonable?

| 1 | 2 | 3 | 4 | 5 |
| --- | --- | --- | --- | --- |
| Not at all acceptable | Slightly acceptable | Moderately acceptable | Very acceptable | Extremely acceptable |

Overall, how satisfied were you with ADepT/CBT?

| 1 | 2 | 3 | 4 | 5 |
| --- | --- | --- | --- | --- |
| Not at all satisfied | Slightly satisfied | Moderately satisfied | Very satisfied | Extremely satisfied |

How likely would you be to recommend CBT/ADepT to friends or family if they needed similar care or treatment?

| 1 | 2 | 3 | 4 | 5 |
| --- | --- | --- | --- | --- |
| Extremely unlikely | Unlikely | Neither Likely or Unlikely | Unlikely | Extremely likely |

Closing remarks:

Anything else you would like to say about ADepT/CBT or taking part in the research?

Debrief

1. Thank client for taking part
2. Cover what will happen next.
3. Let participant know they can e-mail you with follow up comments if they have any further thoughts, giving them a contact e-mail address.

Analytic Method and Stance

The qualitative analysis of the current study was informed by the Framework Method, a highly systematic approach of categorising and organising qualitative data, which falls within the broader family of thematic analysis^1-2^. The Framework Method is not aligned to a specific theoretical framework or approach to qualitative research, and it is intended to be used flexibly and adapted to the purpose and needs of each study. In the current study, an integrative deductive and inductive approach was adopted to ensure that the research questions were addressed adequately, whilst there was also room to explore any unexpected experiences of participants with ADepT. The qualitative analytic stance adopted was a pragmatic post-positivism approach. This approach assumes that there is an objective reality, however, it recognises that this reality cannot be comprehended perfectly, as any researcher’s subjectivity to the analytical process cannot be fully eliminated^3^. KD kept a reflective memoir throughout the analytic process to help identify her own assumptions and put these aside, as much as possible, to minimise the impact of these underlying influencing factors on the results of the current study.

As recommended in reporting standards for reporting qualitative research^4^, KD’s potential sources of bias are reported here for transparency, as identified by the author’s own reflections and in supervision. Salient social and cultural identities of KD are being female, in her mid-twenties, heterosexual, middle class, of Greek Cypriot origin, and at the point of the study having completed an undergraduate degree in psychology and aspiring to gain a place on a doctorate in clinical psychology^5^. These aspects of her identity might have predisposed KD to relate more with participants with similar identities (as for example non-British female participants). Furthermore, her experiences of growing up in a privileged position and within a positive-oriented family context has led her to develop a positive attitude and outlook on life. This means that KD has an inherent passion for positive psychology clinical interventions, as they align with her own life perspective, which could be an additional source of influence. It is also worth also nothing that KD was being supervised for this project by the developer of ADepT (BD), and attending trainings and group therapy supervision with therapists who were learning or currently delivering ADepT. In these supervision and training sessions, KD was exposed to discussions on how therapists worked with ADepT and their views on the treatment protocol.

It is also important to highlight that BD, the primary supervisor of the current study, is the developer of ADepT and NG, the secondary supervisor, is also contributing to the development of depression interventions with a focus on positivity (positive CBT). Furthermore, RW, a qualitative researcher and collaborator on the current study, has a special interest on the experience of anhedonia in adolescent depression. Therefore, it is likely that the supervisors and collaborator of the current study also had specific presumptions about ADepT, which might have influenced the results.

The fact that KD had not met nor interviewed any of the participants in the current study, means that she has not formed any relationships with the participants, which is a common influencing factor in qualitative studies. Although KD has been reflecting on these potential influencers throughout the analytic process, it is likely that these factors still had an impact on the interpretation of data. Therefore, it is hoped that providing this information will help readers judge the validity, relevance, and transferability of the results in their intended context.

In line with the recommended stages of the Framework Method^2^, after the recorded interviews were transcribed (by other members of the research team), KD devoted a substantial amount of time becoming familiarised with the interview transcripts. In this stage, the first 10 interview transcripts were read carefully, whilst a reflective memoir was kept recording first impressions and emerging themes. Specifically, KD used the following four questions to guide her critical and reflective engagement with the data: “what surprises me in this interview?”, “what disturbs me in this interview?”, “what intrigues me in this interview?” and “what is the story behind the story?”. After this stage of becoming immersed in the data, KD open coded the first three interviews, meaning that any data that was deemed to be of importance to the research questions or the general evaluation of ADepT was assigned a representative label. Individual mindmaps were created to visualise the key themes in each of these interviews. Furthermore, one of these interviews was read and open coded by BD, and another interview was read and open coded by RW. KD met with BD and RW separately to discuss each interview and compare and refine the coding schemes, in line with the recommendation of independent coding of initial interviews to enhance the reliability and validity of codes, and robustness of the qualitative analytic procedure^6^. Drawing from these discussions and the coding of the first three interviews, KD formulated an overall working analytical framework, consisting of a hierarchical structure of themes, sub-themes, and codes, to guide the coding of the rest of the interviews. This overall framework was also informed by the research questions, the relevant literature and theoretical background discussed in the Introduction. An ‘other’ category was included in this framework to ensure that any data that initially appeared as irrelevant or not fitting the analytic framework would not be overlooked.

All the interview transcripts were then imported into the NVivo software^7^, to assist in systematically applying the analytical framework to the remaining interviews, by indexing subsequent data to the existing codes and themes. In line with the iterative process of qualitative analysis, the analytic framework was continuously updated as more interviews were coded, and new themes arose. When all interviews had been coded, NVivo was used to generate a framework matrix, i.e. a spreadsheet in which each column represented a code and each row represented a participant, whilst the cells contained the relevant quotes to ensure that the integrity of the original accounts was maintained. This matrix was used to inform the interpretation of data, including identifying the key themes, comparing these between participants and mapping connections between themes. At this stage, theoretical models discussed in the Introduction were also used to frame the data when deemed appropriate. The interpretation of data was intertwined with the write-up of the results. The initial extensive versions of the results were discussed with the supervisors of the current study (BD and NG) who helped refine the final framework to the core themes. In line with the aim of the research, the final framework was formulated with the intention of illustrating the participants’ experiences with and views of ADepT, that are most relevant to informing a definitive trial and the potential implementation of ADepT in routine care settings. Quotes selected for use in the manuscript were double checked to ensure anonymity was preserved for participants, therapists and other individuals referred to.

References

1. Ritchie, J., Spencer, L., & O'Connor, W. *Qualitative Research Practice: A Guide for Social Science Students and Researchers* (2^nd^ Ed.)*.* 2003 London: Sage Publication.
2. Gale, N. K., Heath, G., Cameron, E., et al. Using the framework method for the analysis of qualitative data in multi-disciplinary health research. *BMC Med Res Methodol* 2013; 13: 1-8. <https://doi.org/10.1186/1471-2288-13-117>
3. Guba, E. G., & Lincoln, Y. S. Competing paradigms in qualitative research. In N. K. Denzi, & Y. S. Lincoln (Ed.), *Handbook of Qualitative Research* (pp. 105-117). 1994 Thousand Oaks: Sage Publications.
4. O'Brien, B.C., Harris, I.B., Beckman, T.J. et al. Standards for reporting qualitative research: a synthesis of recommendations. *Acad Med* 2014; 89(9): 1245-1251. <https://doi.org/10.1097/acm.0000000000000388>
5. Jacobson, D., & Mustafa, N. Social identity map: A reflexivity tool for practising explicit positionality in critical qualitative research. *IntJ Qual Methods* 2019; 18: 1-12. <https://doi.org/10.1177/1609406919870075>
6. Mays, N., & Pope, C. Qualitative research: rigour and qualitative research. *BMJ* 1995; 311(6997): 109-112. <https://doi.org/10.1136/bmj.311.6997.109>
7. Bazeley, P., & Jackson, K. *Qualitative data analysis with NVivo* (2^nd^ Ed.). 2013 London: Sage. <https://doi.org/10.1080/14780887.2014.992750>
