# Supplementary material for: A qualitative study exploring depressed participants’ experiences of receiving Augmented Depression Therapy (ADepT)
Source: BMJ Open. 2025 Feb 5;15(2):e088726. doi: 10.1136/bmjopen-2024-088726 (PMC11800203; doi:10.1136/bmjopen-2024-088726)
Supplement: Supplementary file 2 [file bmjopen-2024-088726.reviewer_comments.pdf]

PEER REVIEW HISTORY

BMJ Open publishes all reviews undertaken for accepted manuscripts. Reviewers are asked to complete a checklist review form and are provided with free text boxes to elaborate on their assessment. These free text comments are reproduced below.

ARTICLE DETAILS

Title (Provisional)

A qualitative study exploring depressed participants' experiences of receiving Augmented Depression Therapy (ADepT)

Authors

Demetriou, Kalliopi; Widnall, Emily; Warbrick, Laura; Reed, Nigel; Marchant, Katie; Geschwind, Nicole; Watson, Rebecca; Magner-Parsons, Isabella; Barter, Rachel; Wright, Kim A; dunn, barney

---

VERSION 1 - REVIEW

**Reviewer**	**1**
**Name**	**Lo, Lori Suet Hang**
**Affiliation**	**University of Alberta**
**Date**	**26-Jul-2024**
**COI**	**N/A**

This is a great and important work. Overall, it was well organised and presented. Below are my feedback for improving your manuscript. I hope it would be helpful to you.

Point 1:

- In the introduction, I wonder why ADepT would be your focus among many psychotherapies (p.6 last paragraph). You may consider to explain this earlier or move the content from the last paragraph on p.7 earlier to support your argument.

Point 2:

Please refer to the authors guideline: https://bmjopen.bmj.com/pages/authors#research

- Objectives:It has to be a clear statement of main study aim and major research question.

- Design: Making the sentence more clear would be great.

- Participants: convenience sampled? Did you mention it in the Methods?

- Intervention: I don't think you need to include "intervention" in your abstract as your study is a qualitative study. From the guideline, it says "this can be deleted if there were no interventions.

Point 8:

- Some areas in the Introduction lack references to support (p.6 lines 19-26 and lines 51-55, p.7 lines 10-17 and 20-44, p.8 lines 33-40). Also, inconsistent citation format, i.e. numeric format or APA (p. 8 lines 31, 33)?

Point 10:

- You first mentioned "programme theory" in the research question (p.9 lines- 20-36). You may consider to explain a bit about it earlier in the Introduction. I would ask how this is linked to the recommendations by the MRC? Why is further refinement to the programme theory needed?

- You mentioned "critical realist" was adopted as a stance (p.12 line 52). In my understanding, this critical realist lens stituates between positivism and constructivism. I also wonder why and how you used this lens to inform your analysis, i.e. seeking primarily to explain outcomes, multifactorial causation in an open system? As you suggest to use a "critical realist" stance, I would advise you to explain this idea more.

- In the session of Methods, you described the trial (p. 9 - trial design). You may consider to describe the trial separately in a background of this qualitative study.

- You mentioned about the interview topic guide was used to answer the research questions (p.11 lines 45-56). However, it is unclear how both were linked.

- In the results, adding a sentence summarizing your themes before the subsections would be helpful.

- Your study aim was to explore client views of acceptability, impact, and mechanism of change of ADepT. In the Results, you reported participants quantitatively rated the therapy as acceptable, and presented qualitatively the impacts and mechanisms of the therapy. However, the qualitative data explaining client views of acceptability were not explained explicitly in the Results.

- In the Results (Mechanisms of change in ADepT), I feel like mechanisms could be highlighted in the themes. For example, "establishing a healthier lifestyle; theme 3.3" and "enhanced interpersonal effectiveness; theme 3.7" seem to be short term outcomes of ADepT. In my understanding, mechanisms are the agents of change, such as the reasoning of participants ultimately leading to behaviour changes. From my perspective, possible mechanisms (unseen in the reality) could be participants' increased confidence, motivation, sense of accountability to engage, sense of efficacy, understanding and accepting towards themselves, self-compassion, etc. I would recommend you to define what the meaning of mechanisms of change is and to make the mechanisms explicit in the themes.

- In the Discussion, you mentioned how participant perceptions of mechanism of action of therapy fitted well with the ADEPt logic model. There is a lack of explanation, for example "a

positive bond with the therapist (theme 4.1; logic model box 1)". You may explain more or use a diagram or figure to show how.

- A you mention about MRC and programme theory in the Introduction, you may discuss how your findings can inform future intervention from the perspective of seeing intervention as complexin in this real world, like you concluded in the abstract.

Others:

- A period is missing (p.7 line 51).

Thanks. Keep up the good work!

**Reviewer**	**2**
**Name**	**Välimäki, Maritta**
**Affiliation**	**University of Turku, Department of Nursing Science**
**Date**	**04-Aug-2024**
**COI**	**No competing interests.**

Thank you very much for allowing me to review this manuscript. I read the manuscript with great interest. Effective, easy to use and engaging interventions are needed in mental health area and therefore feedback of end-users are very important. I can also see that authors have put a lot of effort to the study to guide future RCT study.

- The current study explored client views of acceptability, impact, and mechanism of change of ADepT. As authors have described, the original goal of the qualitative study has been changed. In the protocol, it has been stated as follows: “To evaluate feasibility and acceptability, we will examine …..and feedback from patients and therapists about their experience of study participation and therapy” However, due to the promising pilot trial outcomes, the interviews were predominantly with clients who engaged fully with and benefitted from treatment. Authors have also stated that ‘Only around half of individuals treated remit, over half of whom will relapse within two years (Vittengl et al., 2007), meaning a sustained recovery rate of around 25%. Could you clarify your argument in this section why the qualitative study only targeted those patients who were committed to the intervention, not those who discontinued or did not benefit from the intervention. To increase patients’ engagement in the intervention in the future, it is important to understand why the intervention did not work or the reasons for stopping the study early.

- Objective of the study has changed in different part of the manuscript. For example, in the abstract, it has been stated as follows: “The current study explored client views of acceptability, impact, and mechanism of change of ADepT.” In summary: “This study explored participant experience of receiving Augmented Depression Therapy (ADepT) – ….”.

Further, on Page 7: "… to explore their experiences and views of treatment." In addition, the results are reported using a different structure. Can you please ensure that the purpose of the study is expressed similarly in the article and that it is logical with the research questions and results reported.

- The main part in the background describes depression and the intervention at hand. However, what is missing from the background is a description of the conduct of similar types of interviews to access feasibility and acceptability of the new intervention: what previous qualitative studies to assess intervention feasibility or acceptability have been conducted, what do they show, how does this study differ from them, what still needs to be done and why this study needs to be done (a knowledge gap)?

- In abstract, it has been stated as follows: "The current study explored client views of acceptability, impact, and mechanism". In research questions, the following concepts have been used: mechanism of action, implementation issues, contextual moderating factor, feasibility, acceptability, costs and benefit & perceived impact of mechanism of change. Again, in results section, other concepts are reported (impacts, focus on ADept, helpful and unhelpful aspects). So, do the interests of the study coincide in different parts of the manuscript? In addition, which theoretical construction has guided the selection of these above concepts?

- Could you please describe your method section with more details. It is not fully clear what are the inclusion and exclusion criteria for the qualitative study, how the study sample was formed, how the participants were recruited, population size, number of refused etc. For example, what is your rationale that a person who has completed 8 sessions out of 20 has been defined as 'well engaged in the intervention'?

- I understand that it is very easy to describe the methods used in RCT study. However, this paper focuses on the qualitative study and therefore the steps used to describe the methods in your qualitative study are very important. You may want to use some reference to guide your reporting (e.g. Tong et al. 2007, Consolidated criteria for reporting qualitative research (COREQ): a 32-item checklist for interviews and focus groups).

- In generally, the study was assessed to be feasible. I am wondering how feasible the intervention was if it the participants could not finish intervention/data collection after 6 months. As an outcome, the material describes people at completely different stages of the disease or treatment. How this has been taken onboard in analysis or reporting?

- "To ensure maximum sample diversity we monitored on an ongoing basis if our purposive sampling had been successful, and at times made efforts to recruit subgroups that were not represented in the early interviews." Why 'maximum sample diversity' has been emphasized? If diversity of participants was the goal, why were the interview participants not drawn? I understood based on your description that you targeted particularly were those participants who were well engaged in the study/intervention. From this point of view,

the study set should be homogeneous. Please, explain your intention in recruiting the participants.

- Which theoretical construction has guided your interview questions?

- What specific analysis method has been used? Content analysis? Thematic analysis? Mixed methods? Other? I can see that the authors have also used a quantitative approach to analyse and report the results. Is the analysis methods mixed-methods approach? (see examples as follows):

"Less helpful elements of ADept were described by the four participants who only moderately satisfied with treatment (see a discussion section)." "Most participants, ….. some clients' ….(see a results section).

- Results section: "The sample interviewed had a good range in terms of age and gender and were predominantly of White British ethnicity." Why a good range in participants was emphasized? Although it is important to be aware who are our interviewees, a qualitative approach may not necessary seek to generalize the information obtained, but to gain a deeper understanding of the study topic.

- The results section is very long (over 12 pages) and text seems to be very descriptive. The results based on the data categorization is not clear. However, Table 2 is a good summary of the results. Perhaps the result section could be cut and write based on Table 2? It is good to use quotes but the results in text cannot rely on them.

- Discussion: "The current qualitative study explored client perceptions of the acceptability, impacts, and mechanisms of action of ADepT". However, the results have been reported using the following sub-titles: Impacts, Focus on ADept, and Helpful and unhelpful aspects of Adept. Again, are the purpose of the study and results aligned?

- A summary of the results is very long and it repeats the results. How the results differ from previous qualitative studies assessing feasibility and acceptability of interventions? Or are the results similar? What is new in this paper? Deeper reflection of the results is needed.

- "Overall, the present findings suggest that the wellbeing and functional recovery focus of ADepT is acceptable to clients, that a majority of clients experienced meaningful gains during therapy, and that clients' explanations of how this positive change came about largely concurred with the logic model of the intervention." Is there additional evidence to support this claim? Does this describe the real-world situation?

Thank you again for allowing me to review this valuable and timely manuscript. One of the strengths of this article includes users' voice to assess perceptions toward the intervention. This approach provides a valuable view of the topic, allowing for a more nuanced understanding of the impact of interventions on mental health.

VERSION 1 - AUTHOR RESPONSE

Reviewer: 1
Ms. Lori Suet Hang Lo, University of Alberta
Comments to the Author:
This is a great and important work. Overall, it was well organised and presented. Below are my feedback for improving your manuscript. I hope it would be helpful to you.

Author response: We thank the Reviewer for their positive feedback and constructive suggestions about how to further refine the manuscript, which we feel have strengthened the revised submission.

Point 1:
- In the introduction, I wonder why ADepT would be your focus among many psychotherapies (p.6 last paragraph). You may consider to explain this earlier or move the content from the last paragraph on p.7 earlier to support your argument.

Author Response: Thank you for this helpful comment. We have rewritten the rationale to try and be more explicit why we focus on ADepT among many psychotherapies that target positive mental health outcomes. In particular, the focus of ADepT on both symptom relief and wellbeing gain is relatively unique and we now mention earlier it has promising clinical outcomes over the long term:

"A novel psychotherapy,Augmented Depression Therapy (ADepT), has therefore been developed to both reduce depression symptoms and enhance wellbeing and functioning, and shows potential to lead to enhanced treatment outcomes over the longer term[18,20]. ADepT was co-designed alongside experts by experience and clinicians, informed by the intervention mapping framework[21] and the guidance for complex intervention development[22]. While a range of other therapies are being developed to explicitly target positive mental health outcomes across clinical disorders, ADepT is relatively unique in its combined focus on both symptom relief and wellbeing enhancement (a hybrid approach) and its explicit focus on depression[18,23]." (see line 23 p.6 to line 4 p.7).

We cite an in press review that focuses on the range of positive affect treatments that are emerging in literature (new reference 23; Craske et al, in press) that was not included in the original submission.

We have also added in more explicit detail about sustained remission outcomes in ADepT, which indicate it has potential to improve depression treatment outcomes over existing psychotherapies:

"*ADepT led to 80% of clients no longer meeting diagnostic criteria for depression according to clinical interviews at the 6m primary outcome point, and 72% of clients met criteria for sustained remission across all of the 6m, 12m, and 18m assessments. Therefore, ADepT shows potential to enhance long term depression outcomes relative to existing depression psychotherapies."* (see lines 10 – 12, p.8).

This is why we think ADepT is worthy of further empirical examination from both a quantitative and qualitative perspective.

Point 2:
Please refer to the authors guideline: https://bmjopen.bmj.com/pages/authors#research

Author Response: We have now revised the abstract to fully align with these guidelines

- Objectives: It has to be a clear statement of main study aim and major research question.

Author Response: We have rephrased the objectives section of the abstract to be clear what the aim of the study was: "*The current study aimed to explore client views of acceptability, impact, and mechanism of change of Augmented Depression Therapy (ADepT), a novel wellbeing-focused and recovery-oriented psychological therapy for depression.*" (see lines 2-4, p.2)

- Design: Making the sentence more clear would be great.

Author response: This part of the abstract has been revised to read: "*A semi-structured qualitative interview design was used, with data analysed using the framework approach.*" (see lines 5-6, p.2)

- Participants: convenience sampled? Did you mention it in the Methods?

Author Response: Thank you for spotting this inconsistency in the abstract and the main methods. We now simply say 'sampled' in the abstract. Our original plan had been to use purposive sampling to have a mixture of interviews with participants who engaged versus did not engage with treatment and who benefitted from versus did not benefit from treatment. This was not possible to achieve, as the vast majority of participants who completed the trial had benefitted and engaged well with ADepT. We ended up with something closer to convenience sampling – interviewing as many participants in the ADepT arm as we could given research resources and participant availability until we had reached data saturation. We do not have sufficient word count to describe this in the abstract, but describe this in detail in the Methods (see lines 14-26, p.13).

- Intervention: I don't think you need to include "intervention" in your abstract as your study is a qualitative study. From the guideline, it says "this can be deleted if there were no interventions.

Author Response: We have deleted the intervention section from the Abstract as suggested.

Point 8:
- Some areas in the Introduction lack references to support (p.6 lines 19-26 and lines 51-55, p.7 lines 10-17 and 20-44, p.8 lines 33-40). Also, inconsistent citation format, i.e. numeric format or APA (p. 8 lines 31, 33)?

Author Response: We struggled to respond to this comment, as the page and line numbers on our submission version do not fully align with the page and line numbers on the version the Reviewer will have seen. However, we have reread the introduction and checked we are citing relevant studies. In a few places we use multiple sentences to describe the results of key studies (e.g., the Dunn et al., 2023 pilot trial included quantitative and health economic analyses). We cite these studies in the first relevant sentence of a paragraph rather than citing it for every sentence.

The query re citation format we think relates to our use of numeric footnote to refer to footnotes in the original submission. As numeric footnote links of this kind will be confusing alongside Vancouver referencing (see requested change from Editor earlier in this letter), we have removed a majority of footnotes into the main text. The one remaining footnote is labelled a. We are happy to further address this issue if there remain sections the Reviewer wishes us to expand our use of citations.

Point 10:
- You first mentioned "programme theory" in the research question (p.9 lines- 20-36). You may

consider to explain a bit about it earlier in the Introduction. I would ask how this is linked to the recommendations by the MRC? Why is further refinement to the programme theory needed?

Author Response: We have modified the section where we introduce programme theory to try and explain what we mean by programme theory, why iterative updating of programme theory via process evaluation is a good thing in initial development of a treatment, and how qualitative methods assist with this. We also more explicitly reference MRC guidance on process evaluation in this regard:

"The quantitative outcomes are encouraging, but it is also important to evaluate complex interventions like ADepT from a broader qualitative perspective as part of a detailed process evaluation[22,25-28]. This can lead to iterative improvement and refinement of the intervention at early stages of research, to optimise the intervention before conducting definitive clinical trials. Qualitative user feedback can be used to test and update the logic model of the intervention (how an intervention leads to its effects) and enhance understanding of what conditions help or hinder this process (contextual modifying factors), referred to in the UK Medical Research Council process evaluation framework as programme theory[25]. In particular, exploring participants' views of the impact of treatment and how theses impacts were brought about can help identify mechanisms of change in treatment, what intervention elements modulate these mechanisms, and what factors influence the success of these intervention elements in real world settings. These insights in turn can be used to further enhance treatment efficacy. Qualitative methods can also explore participant views on the feasibility and acceptability (defined as "a multi-faceted construct that reflects the extent to which people delivering or receiving a healthcare intervention consider it to be appropriate, based on anticipated or experienced cognitive and emotional responses to the intervention"[29]). Optimising acceptability will increase likely adherence to the intervention and implementation of the intervention both in subsequent definitive trials and in real world implementation after trials[25]." (see line 17 p.8 to line 8 p.9)

This further reading on programme theory also prompted us to reconsider our decision to only present the contextual modifying factors findings in the SOMS in the original submission. Given MRC guidance is explicit about contextual modifying factors being part of programme theory, we now present these findings in the main results. This improves the logical flow of the manuscript for the reader in our view (and also contextualises the recommendation we make in the discussion about considering a readiness assessment at the start of ADepT). This has resulted in the following changes to the manuscript:

a) A new question 4 on contextual modifiers being added to the introduction (lines 9-10, p,10)
b) A new section in the main results on contextual modifiers (entitled barriers and facilitators to engagement with ADepT (line 16 p.27 to line 11 p.29)
c) Review of contextual modifiers briefly in the discussion (lines 10–19, p.32)

The results remain shorter than the original submission even after these additions, as we have condensed description of the original themes as suggested by Reviewer Two (see later in this response letter).

- You mentioned "critical realist" was adopted as a stance (p.12 line 52). In my understanding, this critical realist lens stituates between positivism and constructivism. I also wonder why and how you used this lens to inform your analysis, i.e. seeking primarily to explain outcomes, multifactorial causation in an open system? As you suggest to use a "critical realist" stance, I would advise you to explain this idea more.

Author Response: This was a helpful comment and prompted us to do further reading on critical realism. On reflection, it is more accurate to say we adopted a pragmatic stance, wishing to develop practical understanding of concrete real-world issues that inform action, whilst acknowledging the

impossibility of fully eliminating analyst bias (a post-positivism position). In particular, we wished to further refine the ADepT protocol based on participant feedback.

Moreover, mechanism has a specific meaning in critical realism (generative mechanism interacting at the real level lead to observable but not always observed events at the actual and empirical domain; Mercier, Sanders and Munford et al., 2023, *Int J Qual Methods*) that deviates from how we are using the term to refer to therapeutic mechanism of action of ADepT.

Therefore, this section has been modified to read: "*A pragmatic stance was adopted, aiming to develop practical understanding of concrete, real world issues that can inform action*[37]*, whilst at the same time recognising that an objective reality cannot be comprehended perfectly due to the impossibility of eliminating any researcher's subjectivity to the analytical process (a post-positivist viewpoint)*[38]*.*" (see line 22 p.14 to line 1 p.15)

Please see later response, where we define the pragmatic definition of 'mechanism' we are adopting in the current study.

- In the session of Methods, you described the trial (p. 9 - trial design). You may consider to describe the trial separately in a background of this qualitative study.

Author Response: We have added two new subsection headings to the method ("Background Trial Design", line 17, p.10 and "Qualitative Process Evaluation Design" line 10, p.11) to better differentiate the original trial design and the specific focus in the current manuscript on the qualitative process evaluation component.

- You mentioned about the interview topic guide was used to answer the research questions (p.11 lines 45-56). However, it is unclear how both were linked.

Author response: This was a helpful comment. The topic guide largely followed the earlier one we had used in the ADepT case series (Dunn et al., 2019, BRAT). On reflection, we agree the probes included address some of the aims of the present study more explicitly than others. In particular, it did not include any specific questions about perceived mechanism of action other than positive therapist style. However, the questions and probes were nevertheless sufficient to extract views on these issues. We have added in some additional notes to SOM Section Two that presents the topic guide, explaining how the questions and probes relate directly and indirectly to the three research questions we were focusing on in the present study (see SOM section 2).

In the main methods, we have clarified how the topic guide speaks directly or indirectly to the aims of the study:

"*Interviews followed a topic guide to ensure broad experiences of treatment were covered, which was piloted prior to the study. Acceptability was investigated via questions around views on the wellbeing focus of ADepT and which elements of treatment were seen as helpful or unhelpful. Impacts were identified via a series of questions of immediate and longer-term consequences of treatment. Contextual modifying factors were explored via questions around barriers and facilitators to engagement with ADepT. Given the centrality of positive therapist style to the ADepT logic model , a series of questions explored participants views on the positive, solution-focused, and future-oriented therapist style of ADepT and how this impact on their relationship with the therapist (i.e. formation of a positive emotional bond). There were no other explicit questions about participants' views on mechanism of action of ADepT, but these themes spontaneously emerged in response to other*

questions. There were also questions exploring views on engagement with the research process, which are not the focus of the current study and so are not considered further here. Interviewers were also encouraged to explore any additional issues or themes that emerged in the interviews. The topic guide was continuously updated based on incoming data during interviews." (see line 18 p.12 to line 5 p.16)

- In the results, adding a sentence summarizing your themes before the subsections would be helpful.

Author response: We agree and now introduce a sentence at the start of each theme that summarizes the subthemes that will be explored. Each subtheme then also begins with a summary (see Results p.16 to p.29).

- Your study aim was to explore client views of acceptability, impact, and mechanism of change of ADepT. In the Results, you reported participants quantitatively rated the therapy as acceptable, and presented qualitatively the impacts and mechanisms of the therapy. However, the qualitative data explaining client views of acceptability were not explained explicitly in the Results.

Author Response: Thank you for this very helpful comment, which echoes similar points made by the second Reviewer about a lack of consistency in how we present the aims of the study and then order the results (and also inconsistent use of language relating to study aims and objectives in different parts of the manuscript). On reflection we realise that there was a mismatch: The rationale stated that we were assessing acceptability via views on wellbeing focus and helpful vs unhelpful aspects of treatment *("What were clients' views about the feasibility and acceptability of ADepT, in particular the focus on enhancing wellbeing versus reducing depression and which elements of the intervention did they find helpful or unhelpful?", question 1, p.9).* However, we did not explicitly mention in the results that these sections spoke to acceptability and had placed these two elements of acceptability in different sections. In the revised manuscript results, we now present these two themes alongside each other and are explicit they relate to acceptability:

"*Acceptability of therapy was explored in terms of views on the wellbeing focus of ADepT and perceived helpful and unhelpful elements of treatment . …… "* (see line 5-6, p. 18)

We have revised language in all sections to be consistent in the terms we use to refer to the study objectives and how our findings speak to them.

- In the Results (Mechanisms of change in ADepT), I feel like mechanisms could be highlighted in the themes. For example, "establishing a healthier lifestyle; theme 3.3" and "enhanced interpersonal effectiveness; theme 3.7" seem to be short term outcomes of ADepT. In my understanding, mechanisms are the agents of change, such as the reasoning of participants ultimately leading to behaviour changes. From my perspective, possible mechanisms (unseen in the reality) could be participants' increased confidence, motivation, sense of accountability to engage, sense of efficacy, understanding and accepting towards themselves, self-compassion, etc. I would recommend you to define what the meaning of mechanisms of change is and to make the mechanisms explicit in the themes.

Author comment: This was an insightful point and we have reflected on how we can more clearly differentiate outcomes and mechanisms in complex interventions like ADepT. In our view, there is often a complex interaction between changes in different parts of a presentation, such as improvement

in one area (interpersonal functioning) can then lead to changes in another areas (such as mood) or vice versa. In this sense, each process is both an outcome (a valued change resulting in therapy in its own right) and a mechanism (somethings that then drives change in another valued outcome). In mental health interventions, there is also an equal weighting of importance of affective, bodily, cognitive and behavioural change (rather than affective, body and cognition simply being mechanisms to drive behavioural change). Moreover, we definitely did not mean to use ‘mechanism’ in the specific way it is referred to in critical realism (see earlier response). Therefore, we have decided to define and adopt a heuristic distinction between outcome and mechanism in the manuscript:

“The distinction between what is an impact of treatment and what is a mechanism of treatment is not clear-cut. For example, initial change in interpersonal communication style could be seen as an early impact, which in turn could lead to subsequent improvement in mood (and hence is also a mechanism of change). Here we use impact to refer to changes in the high-level objectives of ADepT to enhance wellbeing and functioning over the long time. We use mechanism to refer to any change that occurs during therapy that helps bring this about, which may or may not be a more a proximal impact in its own right (including changes in thinking, feeling, body, and behaving).” (see footnote a, p.35)

- In the Discussion, you mentioned how participant perceptions of mechanism of action of therapy fitted well with the ADEPt logic model. There is a lack of explanation, for example "a positive bond with the therapist (theme 4.1; logic model box 1)". You may explain more or use a diagram or figure to show how.

Author response: We agree this section was too telegraphic and did not fully explain the links between the themes in the mechanisms section and the logic model of the intervention. We have added the following section to the revised manuscript to make this more explicit:

“Regarding mechanism aims, participant perceptions of the impact and mechanisms of action of therapy fitted well with the ADepT logic model (Figure1). Participants described the benefits of a positive therapist bond (theme 2.1) and how ADepT helped them attend more to positives in a solution-focused fashion (theme 4.1), aligning with the emphasis in the logic model of building and sustaining a positive relationship with the participant to cultivate a solution-focused outlook (Box 1 in Figure 1). Participants reported that clarifying values and working towards values consistent goals was beneficial, consistent with the emphasis on values exploration and behavioural activation towards values consistent goals in the logic model (Box 2 and 3 in Figure 1), and supporting the emphasis on values work in ADepT. Participants described having enhanced capacity to both maximise the impact of life opportunities to build wellbeing and positive emotions, and to minimise the impact of life challenges on depression and negative emotions (theme 3.1 and 3.2). This aligns with the logic model’s emphasis on building both resilience and thriving (Box 5a and 5b in Figure 1) and also with ADepT’s theoretical emphasis on both down-regulating the negative valence system and up-regulating the positive valence system[18,47]. Participants described how ADepT gave them skills to notice and respond differently to unhelpful depressogenic habits (theme 4.6), concordant with the emphasis on opposite action in the logic model (Box 4 in Figure 1). Participants commented on having a new relationship with depression (theme 4.7) and feeling they have more proactive control of life (theme 4.5), which accords with the emphasis on changing relationship to depression and enhanced self-efficacy to manage it in the logic model (Box 6 in Figure 1). Participants described benefitting from booster sessions to remain well and continuing their progress to values-consistent goals. This also aligns with the logic model’s emphasis on wellbeing-planning for the future (Box 6, Figure 1).”(see line 14 p.31 to line 9 p.32)

- A you mention about MRC and programme theory in the Introduction, you may discuss how your findings can inform future intervention from the perspective of seeing intervention as

complex in in this real world, like you concluded in the abstract.

Author Response: As discussed above, we have added the contextual modifiers section from the SOM to the main manuscript in the revision, which speaks more explicitly to what impacts on implementation in the real world context (see p.27 to p.29)

We comment on this in the discussion:

"Regarding contextual modifying factor aims, key facilitators to engagement with ADepT were readiness and openness to change when starting therapy, having a supportive social network, and therapist flexibility around scheduling. Potential barriers to engagement were having significant life challenges (too much going on, poor physical health, and/or major negative life events), particularly when therapy tools were not perceived as being able to help modulate or influence these external factors. These are similar to barrier and facilitator themes that emerged in qualitative studies of Behavioural Activation and CBT for depression[47]. While these issues are not unique to ADepT, they illustrate the importance of therapists acknowledging and working with the 'real-world context' their clients are operating in to maximise their engagement with and benefit from treatment[22,25]." (see line 10-9, p.32).

Others:
- A period is missing (p.7 line 51).

Author response: As before, our page and line numbering does not perfectly align with the version the Reviewer has seen. However, on rereading the manuscript we found one missing period we have now corrected. Thanks for spotting this typo.

Thanks. Keep up the good work!

Author response: Thanks again for positive comments and constructive suggestions for improvement!

Reviewer: 2
Prof. Maritta Välimäki, University of Turku
Comments to the Author:
Thank you very much for allowing me to review this manuscript. I read the manuscript with great interest. Effective, easy to use and engaging interventions are needed in mental health area and therefore feedback of end-users are very important. I can also see that authors have put a lot of effort to the study to guide future RCT study.

Author: We thank the Reviewer for positive feedback on the focus of the manuscript and for a detailed set of suggested improvements to the manuscript.

- The current study explored client views of acceptability, impact, and mechanism of change of ADepT. As authors have described, the original goal of the qualitative study has been changed. In the protocol, it has been stated as follows: "To evaluate feasibility and acceptability, we will examine .....and feedback from patients and therapists about their experience of study participation and therapy" However, due to the promising pilot trial outcomes, the interviews were predominantly with clients who engaged fully with and benefitted from treatment.

Author response: Thank you for this helpful comment, which has prompted us to be more explicit about the objectives of the study and the methods by which we achieved them in the revised

manuscript. In our view, the goal of the study did not change at any point (we had always planned to explore participant experience around themes of acceptability, impact, mechanisms and implementation of ADepT; see Dunn et al., 2019 Pilot and Feasibility Studies, p.9).

However, on reflection, we realised we had not been consistent in the use of language around these objectives across the manuscript and the order in which we presented the results to address these questions (see earlier response to Reviewer One). We now present acceptability, impact and mechanism findings in the results in an order that aligns with the order in which we introduced the questions in the rationale.

In our view, clarifying study objectives is a somewhat separate issue from the sampling method we used to achieve these goals. Our plan had been to use purposive sampling to get a mix of levels of engagement and outcomes from ADepT. However, this was not possible as in the trial a vast majority of clients engaged well with and benefitted from ADepT. Therefore, we instead moved to more pragmatic convenience sampling, interviewing participants until we reached data saturation and maximising available research resources to achieve this. We discuss this change in sampling approach in detail in the methods (see line 14 – 26, p.13).

Authors have also stated that 'Only around half of individuals treated remit, over half of whom will relapse within two years (Vittengl et al., 2007), meaning a sustained recovery rate of around 25%. Could you clarify your argument in this section why the qualitative study only targeted those patients who were committed to the intervention, not those who discontinued or did not benefit from the intervention. To increase patients' engagement in the intervention in the future, it is important to understand why the intervention did not work or the reasons for stopping the study early.

Author response: This was a helpful comment in encouraging us to be more explicit in the rationale when we are describing outcomes from existing psychotherapies versus ADepT. The section of the rationale the Reviewer is referring to describe outcomes from existing gold standard psychotherapies like CBT for treatment (<50% remission at end of acute treatment; only 25% sustained remission at longer term follow-up), which is what motivated us to develop ADepT in the first place to attempt to improve these sub-optimal long term treatment outcomes. The pilot trial of ADepT shows more promising results, with higher rates of acute and sustained remission. Therefore, it is not that the current qualitative study solely targeted those patients who were committed to the intervention or benefitted from it. It is simply that the sample in the trial who were available to us predominantly did engage and benefit from the treatment - we interviewed the sample we had available to us. We agree it would have been useful to interview the five people who dropped out of the intervention and trial early, but as they had withdrawn from the research project it would not have been ethical to approach them to do so. As discussed in the methods, we made particular efforts to attend to the handful of people interviewed who had not engaged and/or fully benefitted from the treatment in our results and discussion to try and capture the voices of those who were less satisfied. We have made a number of changes to the introduction to communicate this point more clearly, in particular that sustained remission rates of standard therapies are relatively disappointing and look more promising in ADepT:

"Less than 50% of individuals treated with current gold-standard evidence-based therapies like cognitive behavioural therapy (CBT) respond or fully remit[5-6], more than half of whom will relapse within two years[7], meaning a sustained recovery rate of around 25%." (see lines 5-7, p.6)

" *ADepT led to 80% of clients no longer meeting diagnostic criteria for depression according to clinical interviews at the 6m primary outcome point, and 72% of clients met criteria for sustained*

remission across all of the 6m, 12m, and 18m assessments. Therefore, ADepT shows potential to enhance long term depression outcomes relative to existing depression psychotherapies." (see lines 10 – 14, p.8)

- Objective of the study has changed in different part of the manuscript. For example, in the abstract, it has been stated as follows: "The current study explored client views of acceptability, impact, and mechanism of change of ADepT." In summary: "This study explored participant experience of receiving Augmented Depression Therapy (ADepT) – ....". Further, on Page 7: "... to explore their experiences and views of treatment." In addition, the results are reported using a different structure. Can you please ensure that the purpose of the study is expressed similarly in the article and that it is logical with the research questions and results reported.

Author response: This was an extremely helpful comment. As discussed above, while the aims of the study have not changed in different parts of the manuscript in our view, we agree we could have presented findings in a more logical order to align to our principal research questions. Please see earlier responses to Reviewer One as to how this has been corrected so that the structure of the article logically addresses each research question in turn (acceptability, impacts, mechanism, and contextual modifying factors) and uses consistent language throughout the manuscript.

- The main part in the background describes depression and the intervention at hand. However, what is missing from the background is a description of the conduct of similar types of interviews to access feasibility and acceptability of the new intervention: what previous qualitative studies to assess intervention feasibility or acceptability have been conducted, what do they show, how does this study differ from them, what still needs to be done and why this study needs to be done (a knowledge gap)?

Author Response: We agree we were not explicit in the original rationale as to how this study builds on previous qualitative work on ADepT. We have added the following section to the introduction to briefly describe preliminary qualitative findings from the earlier case series (Dunn et al., 2019) and to articulate why further qualitative investigation is needed:

"There has been only limited qualitative exploration of participants' experience of ADepT to date. Thematic analysis of interviews with eleven participants who took part in the original case series on an early version of the ADepT protocol[20] *found that the wellbeing focus of treatment was acceptable, that treatment led to meaningful gains in wellbeing and reductions in depression, and that ADepT helped changed their relationship to ongoing depression so that they were more able to be resilient when mood was low. Helpful elements of therapy were the positive therapeutic alliance that was established, clarifying values, enhancing engagement with simple everyday pleasures, and optimising self-care. While therapy homework exercises were experienced as on balance helpful, for some participants this felt overwhelming. Given the small sample used in that case series*[20]*, saturation was not reached. This qualitative analysis also did not explore in detail perceived mechanisms of change. Moreover, given the iterative nature of intervention development, it is important to further explore views of participants who underwent the updated protocol used in the subsequent pilot trial*[18,20].

To address this gap, the current study makes use qualitative interviews with participants taking part in the trial that explored their experiences and views of treatment[24]*."* (see lines 9-23, p.9)

- In abstract, it has been stated as follows: "The current study explored client views of acceptability, impact, and mechanism". In research questions, the following concepts have

been used: mechanism of action, implementation issues, contextual moderating factor, feasibility, acceptability, costs and benefit & perceived impact of mechanism of change. Again, in results section, other concepts are reported (impacts, focus on ADept, helpful and unhelpful aspects). So, do the interests of the study coincide in different parts of the manuscript? In addition, which theoretical construction has guided the selection of these above concepts?

Author response: Please see earlier responses as to how this has been corrected so that the structure of the article logically addresses each research question in turn and uses consistent language throughout the manuscript. In terms of the theoretical construction guiding selection of these concepts, this is largely following MRC guidance on complex intervention development and how to embed process evaluation into trials, which we discuss in the introduction:

"The quantitative outcomes are encouraging, but it is also important to evaluate complex interventions like ADepT from a broader qualitative perspective as part of a detailed process evaluation[22,25-28]." (see lines 17-19, p.8).

- Could you please describe your method section with more details. It is not fully clear what are the inclusion and exclusion criteria for the qualitative study, how the study sample was formed, how the participants were recruited, population size, number of refused etc. For example, what is your rationale that a person who has completed 8 sessions out of 20 has been defined as 'well engaged in the intervention'?

Author Response: We have checked and revised where appropriate the methods section to make sure all of these elements are covered (see also response to later suggestion to use COREQ checklist). We have added additional information where needed:

Methods:

"*82 adults experiencing a current major depressive episode according to the structured clinical interview for depression (SCID-I)[30], scoring > 10 on the Patient Health Questionnaire (PHQ-9)[31], and with anhedonic features (scoring at least 2 on item one of the PHQ-9 measuring anhedonia [i.e, more than half the days of the past two weeks]) were recruited, primarily from local NHS Talking Therapy high-intensity waiting lists."* (lines 21-25, p.10)

"*All participants recruited into the trial were eligible to take part in these interviews and all initially consented to be contacted at a later point to schedule them (although we explained at the point of trial recruitment that only a subset of participants who consented would eventually be approached for the interviews). We focus solely here on interviews with participants in the ADepT arm.*

Participants were approached to schedule the interview by the preferred method of contact they had given when initially recruited into the trial (either telephone, e-mail, letter and/or or video conferencing). If participants could not be contacted by this method, other methods they had given permission for where then also tried. Three attempts were made to book an interview with participants – if after this time no reply had been received, it was assumed they no longer wished to take part" (lines 13-23, p. 11).

Results: "*Of 41 participants recruited into the ADepT arm, five participants dropped out of the research before the interview phase, leaving a possible pool of 37 participants. Twenty participants were contacted, continued to consent to the interview, and were interviewed (49% of individuals treated with ADepT in the trial). A further three participants were contacted, but no longer consented to take part in the interview component of the research (two said they were too busy and one said she would struggle with spoken English at it was her second language). We were unable to contact eight participants. We did not attempt to contact the remaining six participants, as data saturation had been reached and the trial was coming to end."* (line 18 to 25, p.16)

Appendix One and Two at the end of this manuscript show where all requirements of both SRQR and COREQ checklists are incorporated into the manuscript.

Regarding minimum adequate dose, a common rule of thumb in psychotherapy research is to use 50% of the maximum intervention dose to indicate satisfactory engagement (e.g. in previous MBCT trials run in our centre; Kuyken et al., 2015 PREVENT trial, Lancet). The acute phase of ADepT is 15 sessions, so 8 sessions means at least 50% of the acute dose has been completed. We pre-specified this in the original trial protocol. We articulate the 50% of acute dose rule of thumb in the methods:

"The original intention had been to use purposive sampling to recruit a representative subset of participants who did versus did not complete a minimum adequate dose of ADepT (at least 50% of the acute therapy dose; 8 sessions)" (see lines 14-17, p.13)

- I understand that it is very easy to describe the methods used in RCT study. However, this paper focuses on the qualitative study and therefore the steps used to describe the methods in your qualitative study are very important. You may want to use some reference to guide your reporting (e.g. Tong et al. 2007, Consolidated criteria for reporting qualitative research (COREQ): a 32-item checklist for interviews and focus groups).

Author response: We agree on the importance of method description in qualitative research. As per journal requirements, our reporting was informed by the 'Standards for Reporting Qualitative Research' (SRQR) guidance checklist. This checklist was included on the final page of the original submission (p.52). However, we did not explicitly acknowledge this in the methods. We apologise for this oversight.

As COREQ has some differences from SRQR, for the sake of completeness, we now use both checklists and ensure all required information is included in the manuscript. There is no clear preference for using one checklist over the other as far as we are aware (see Peditto et al., 2018, HERD, 10.1177/1937586718772615).

We have added the following line to the methods:

"The conduct and reporting of this qualitative process analysis is informed by the Standards for Reporting Qualitative Research[33] and the Consolidated Criteria for Reporting Qualitative Studies[34]." (see lines 16-18, p.14).

We include both checklists an appendix to this letter. As the page numbers refer only to the submission version of the manuscript (and will change in the final proofs if the article is accepted), we have not included them in the SOMS.

- In generally, the study was assessed to be feasible. I am wondering how feasible the intervention was if it the participants could not finish intervention/data collection after 6 months.

Author Response: Study feasibility was assessed in our earlier paper, which evaluated pre-specified continuation criteria (Dunn et al., 2023, Lancet EClinical Medicine). All of these criteria were fully met. We agree with the Reviewer however that one learning point regarding study design is to consider having a slightly later primary endpoint in a later definitive trial (i.e., at eight months a greater proportion of participants would have completed acute treatment). This is a hard balance to get right: if this outcome point is set too early, many participants will not have completed treatment; if it is set too late, many participants will have had an extensive gap between end of treatment and outcome assessment.

The focus of the present qualitative study was on intervention feasibility not on study feasibility. At the design stage of the intervention, experts by experience told us they would appreciate flexible scheduling of acute sessions to accommodate life issues (e.g. allowing breaks in scheduling for illness, holidays, childcare) and the incorporation of an optional booster phase for a year after treatment (to support long term relapse prevention in depression). Therefore, there was never a strict intention to complete treatment within six months. Evidence of intervention feasibility are strong rates of engagement with ADepT (with the vast majority completing the full dose and very few dropping out), that participants gave positive quantitative ratings of acceptability, and that interviews revealed that participants found treatment acceptable and described a wide range of positive impacts.

As the present study focuses on intervention feasibility, not study feasibility, we have not commented on the primary outcome assessment point issue in the revised manuscript.

As an outcome, the material describes people at completely different stages of the disease or treatment. How this has been taken onboard in analysis or reporting?

Author response: In terms of disease stage, we did not assess depression status at the point each qualitative interview was conducted. However, we now report remission status at each of the 6m, 12m and 18m assessment in SOM Section One. At 6m, eighteen of twenty participants interviewed had remitted. Sustained remission at each of the 6m, 12m and 18m assessment was shown by thirteen of the nineteen individuals where complete data were available. Of the remaining six individuals, one (AD88) did not remit at any assessment, one (AD32) only remitted at 6m, and the remaining four were in remission at two out of three assessments. The one participant who did not complete the 18m assessment (AD18) was in remission at both the 12m and 18m assessment. This suggests relatively little variance in disease stage, with most participants remaining well for all or a majority of follow-up assessments. We have added the following line to the description of participants in the results:

"Eighteen of twenty participants no longer met diagnostic criteria for depression on clinical interview (i.e., were in remission) at the six-month primary outcome. Nineteen participants had diagnostic data at each of the 6m, 12m and 18m assessment. Of these, thirteen met remission criteria at all time points. Only one participant (AD88) did not meet remission criteria at any assessment. Therefore, the sample consisted predominantly of individuals who showed sustained benefits from ADepT." (see lines 10 to 15, p.17)

In terms of treatment stage, all participants had completed acute treatment when they were interviewed. However, there was variability in length of time since completing acute treatment (which meant some were still in the optional booster phase whereas others had completed it at the point they were interviewed). We include data on time between completing acute treatment (in months) and completing the interview (and if the interview was conducted during or after the booster phase) in SOM Section One. No obvious patterns as a function of time since interview and booster stage status emerged in the data during analysis, but nevertheless we now explicitly mention this as a limitation:

"*It is conceivable that participants' views may have been modulated by whether or not they were still in the booster phase and that their memory of the intervention might have been limited for those interviewed a long time after treatment was completed. However, there was no discernibly different pattern of responses given as a function of treatment stage, and only one participant commented that they struggled to remember their experiences with therapy)."* (see lines 21-25, p. 33)

- "To ensure maximum sample diversity we monitored on an ongoing basis if our purposive sampling had been successful, and at times made efforts to recruit subgroups that were not represented in the early interviews." Why 'maximum sample diversity' has been emphasized? If diversity of participants was the goal, why were the interview participants not drawn? I

understood based on your description that you targeted particularly were those participants who were well engaged in the study/intervention. From this point of view, the study set should be homogeneous. Please, explain your intention in recruiting the participants.

Author response: We did not intentionally target individuals who engaged well with and benefitted from ADepT – it is simply that the pool of participants available to us from the trial had predominantly engaged well with and benefitted from the approach. We have removed the sentence above, as while we did monitor on an ongoing basis if purposive sampling was going according to plan, our efforts to recruit subgroups of non-responders and partial engagers was not successful for the reasons outlined above.

- Which theoretical construction has guided your interview questions?

Author response: We based our interview questions on the topic guide used in our earlier case series paper (Dunn et al., 2019a BRAT). This in turn was derived from the topic guide used in the earlier COBRA trial run in our centre (a comparison of BA and CBT that included an interview component; Finning et al., 2017 https://doi.org/10.1136/bmjopen-2016-014161). Our experience is that this topic guide had a series of questions and prompts that were sufficient to allow clients to elaborate on their experience. Please also see our earlier response to Reviewer One regarding how the interview questions aligned to the focus of the current study. To avoid further lengthening the manuscript, we have not included this information in the manuscript – we are happy to reconsider this if the Reviewer feels it is essential.

- What specific analysis method has been used? Content analysis? Thematic analysis? Mixed methods? Other? I can see that the authors have also used a quantitative approach to analyse and report the results. Is the analysis methods mixed-methods approach? (see examples as follows):
"Less helpful elements of ADept were described by the four participants who only moderately satisfied with treatment (see a discussion section)." "Most participants, ... some clients' ....(see a results section).

Author response: As described in the method (and elaborated on in detail in SOMS) of the original submission, our analysis used the Framework Approach, a form of thematic or content analysis (see Gale et al., 2013, BMC Medical Research Methods). This was supplemented with some mixed method elements (in particular using treatment satisfaction ratings and treatment outcomes to identify individuals who had benefitted less from ADepT and ensure these voices were represented in the analysis). We are happy to provide further details if the Reviewer feels this would be beneficial.

- Results section: "The sample interviewed had a good range in terms of age and gender and were predominantly of White British ethnicity." Why a good range in participants was emphasized? Although it is important to be aware who are our interviewees, a qualitative approach may not necessary seek to generalize the information obtained, but to gain a deeper understanding of the study topic.

Author response: We have modified the language to simply describe the sample, not to comment on the adequacy of the range observed:

"*The sample interviewed were a mixture of genders and ages and were predominantly of White British ethnicity….*" (see lines 2-3 p.17)

- The results section is very long (over 12 pages) and text seems to be very descriptive. The results based on the data categorization is not clear. However, Table 2 is a good summary of the results. Perhaps the result section could be cut and write based on Table 2? It is good to use quotes but the results in text cannot rely on them.

Author response: We appreciate the positive feedback on Table Two being a helpful summary. We have condensed the reporting of the original qualitative findings by 25% (original submission 4034 words, revised submission comparable sections 3130 words), whilst still attempting to provide an account of the themes that goes beyond mere description and does not rely solely on the quotes presented. This is broadly comparable in length to results of similar qualitative studies we have seen reported in the journal (e.g. Finning et al., 2017 on COBRA trial). To make it clearer how the results section aligns to Table 2, we include a summary of each theme at the beginning of each section (as recommended by Reviewer One). We hope also that reordering the results and being clear how each section speaks to acceptability, impact, and mechanisms aims of the study makes it clearer how results align with our data categorization. We are happy to make further changes if the Reviewer comments on specific sections where it remains unclear how the results relate to the overarching theme or linked quotes.

As discussed in Response to Reviewer One, we have moved the contextual modifying section from the SOMS to the main manuscript, as this aligns more with MRC definition of programme theory and makes the findings more logically coherent. Even taking this addition into account, we have made the revised results section more concise..

- Discussion: “The current qualitative study explored client perceptions of the acceptability, impacts, and mechanisms of action of ADepT”. However, the results have been reported using the following sub-titles: Impacts, Focus on ADept, and Helpful and unhelpful aspects of Adept. Again, are the purpose of the study and results aligned?

Author response: See earlier response about how we have now aligned study aims, study results, and study discussion regarding the acceptability, impact and mechanism questions.

- A summary of the results is very long and it repeats the results. How the results differ from previous qualitative studies assessing feasibility and acceptability of interventions? Or are the results similar? What is new in this paper? Deeper reflection of the results is needed.

Author response: We have condensed the summary of the results in the discussion. We have also realigned the discussion to discuss the primary acceptability, impact, mechanisms and modifying objectives of the paper in turn. The key implication of the present paper is ADepT is acceptable, results in positive impacts, works largely in the way it is intended to, and we have gained insights into what helps and hinders engagement with it. This, in combination with the promising quantitative results of the pilot trial, warrants continuation to definitive trial. To further promote deeper reflections on the findings, in the revised discussion we now cross-reference back to how these findings build on the preliminary qualitative work from the ADepT case series (Dunn et al., 2019, BRAT), the only previous study of client experience of ADepT. We also discuss how findings relate to other qualitative work on related interventions like BA and positive CBT, and how findings support broader themes emerging in the positive psychology and literature. The main action output intended from this study (consistent with the pragmatic focus in the Framework Method) was to identify further enhancements to ADepT, which we outline at the end of the discussion:

"The present findings suggest a number of minor modifications that could be made to the ADepT logic model and intervention delivery to further enhance the intervention. Most importantly, some modifications are needed to ensure that therapists remain in participants' 'zone of proximal development' for their current mood state, including adjusting current action steps and goals as mood state fluctuates within and between sessions. The ADepT rationale presented to participants could be explicit about the need to modulate expectations and to pace levels of activation as a function of current mental state (i.e., adapting 'stretch'). Further, therapists need to more explicitly monitor for and respond to self-criticism being triggered when progress is not at participants' desired pace. Suggesting areas for logic model refinement, participants also identified as key mechanisms of change enhancing interpersonal skills (assertiveness and help seeking) and building self-care and self-compassion, both of which are relatively implicit in the current programme logic. The importance of enhancing hope as an outcome of treatment also suggests hope could be included more explicitly as an outcome target in the ADepT logic model. Treatment engagement could be enhanced by integrating more explicit 'readiness assessments' as used in dialectical behaviour therapy at the start of treatment (and, if required, engage in some pre-commitment work to build motivation, recruit external support, and problem-solve external life challenges to scaffold treatment engagement)[48].

Other suggestions for protocol refinement that emerged included simplifying tools used in therapy, being more explicit about the intended function of each session at the start to avoid drift, and setting home practice expectations more clearly. A number of participants said they would have benefitted from ongoing support after booster treatment had finished, for example through peer led drop-in support groups and access to a digital resource hub." (see line 19 p.32 to line 15 p. 33).

- "Overall, the present findings suggest that the wellbeing and functional recovery focus of ADepT is acceptable to clients, that a majority of clients experienced meaningful gains during therapy, and that clients' explanations of how this positive change came about largely concurred with the logic model of the intervention." Is there additional evidence to support this claim? Does this describe the real-world situation?

Author response: As discussed in the previous point, other evidence for acceptability, impact and mechanism of ADepT comes from promising initial quantitative and qualitative findings from the case series (Dunn et al., 2019, BRAT) and the quantitative findings of the pilot trial (Dunn et al., 2023, Lancet Eclinical medicine). We also have recently published a study evaluating whether similar findings emerge when training routine therapists in routine settings to deliver ADepT (i.e. the 'real-world' situation; Warbrick et al., 2024). We briefly comment on this in the revised discussion:

"*Fifth, existing work evaluating ADepT has been conducted with participants who received ADepT in a university research clinic. It is important to consider if these positive findings will also emerge in routine care settings. A recent pilot implementation study showed that therapists in routine NHS Talking Therapy settings could be trained to deliver ADepT with fidelity and competence, that this led to positive treatment outcomes with their clients, and that therapists felt the model was effective and a good fit for NHS Talking Therapies settings[51]. This provides preliminary evidence that effects are likely to hold in 'real-world' settings."* (see lines 9 to line 15, p. 34)

Thank you again for allowing me to review this valuable and timely manuscript. One of the strengths of this article includes users' voice to assess perceptions toward the intervention. This approach provides a valuable view of the topic, allowing for a more nuanced understanding of the impact of interventions on mental health.

Author Response: We would like to thank the Reviewer for their detailed critique and positive feedback, and we feel the manuscript has been improved by incorporating their suggestions.

*** ***

COI statements:

Reviewer: 1
Competing interests of Reviewer: N/A.

Reviewer: 2
Competing interests of Reviewer: No competing interests.

Appendix One:

Standards for Reporting Qualitative Research (SRQR) Guidance Checklist

Item	Page
**Title and Abstract**	
Item 1. Title: Concise description of the nature and topic of the study. Identifying the study as qualitative or indicating the approach (e.g., ethnography, grounded theory) or data collection methods (e.g., interview, focus group) is recommended	Y - p.1, line 1-2
Item 2. Abstract: Summary of key elements of the study using the abstract format of the intended publication; typically includes background, purpose, methods, results, and conclusions.	Y - p.2, line 1 -24
**Introduction**	
Item 3. Problem Formulation: Description and significance of the problem/phenomenon studied; review of relevant theory and empirical work; problem statement.	Y - p.6 – p.11
Item 4. Purpose or research question: Purpose of the study and specific objectives or questions.	Y – line 23 p.9 to line 4 p.10
**Methods**	
Item 5. Qualitative approach and research paradigm: Qualitative approach (e.g., ethnography, grounded theory, case study, phenomenology, narrative research) and guiding theory if appropriate; identifying the research paradigm (e.g., post-positivist, constructivist/interpretivist) is also recommended; rationale.	Y line 20 p.14 – line 1 p.15
Item 6. Researcher characteristics and reflexivity: Researchers' characteristics that may influence the research, including personal attributes, qualifications/experience, relationship with participants, assumptions, and/or presuppositions; potential or actual interaction between researchers' characteristics and the research questions, approach, methods, results and/or transferability.	Y – SOM Section 3
Item 7. Context: Setting/site and salient contextual factors; rationale.	Y – line 14 p.10 to line 6, p.11
Item 8. Sampling strategy: How and why research participants, documents, or events were selected; criteria for deciding when no further sampling was necessary (e.g., sampling saturation); rationale.	Y – line 10-20, p.11; line 11-24 p.13

Item 9. Ethical issues pertaining to human subjects: Documentation of approval by an appropriate ethics review board and participant consent, or explanation for lack thereof; other confidentiality and data security issues.	Y – line 11 to line 18 p.14
Item 10. Data collection methods: Types of data collected; details of data collection procedures including (as appropriate) start and stop dates of data collection and analysis, iterative process, triangulation of sources/methods, and modification of procedures in response to evolving study findings; rationale.	Y - p.11 – p.14 (also SOMS Section 2 and Section 3)
Item 11. Data collection instruments and technologies: Description of instruments (e.g., interview guides, questionnaires) and devices (e.g., audio recorders) used for data collection; if/how the instrument(s) changed over the course of the study	Y- p.11 – p.14 SOMS S2
Item 12. Units of study: Number and relevant characteristics of participants, documents, or events included in the study; level of participation.	Y – line 17 p.16 to line 17 p.17; Table 1 p.44; SOM Table 1
Item 13. Data processing: Methods for processing data prior to and during analysis, including transcription, data entry, data management and security, verification of data integrity, data coding and anonymization / de-identification of excerpts.	Y - p14 to 16, SOM Section 3
Item 14. Data analysis: Process by which inferences, themes, etc. were identified and developed, including the researchers involved in data analysis; usually references a specific paradigm or approach; rationale.	Y - p14 to 16, SOM Section 3
Item 15. Techniques to enhance trustworthiness: Techniques to enhance trustworthiness and credibility of data analysis,(e.g., member checking, triangulation, audit trail); rationale.	Y line 18 -20 p.15; line 4 – 8, p. 34
**Results/Findings**	
Item 16. Synthesis and interpretation: Main findings (e.g., interpretations, inferences, and themes); might include development of a theory or model, or integration with prior research or theory.	Y - p.16 – p.29, Table 2 p. 45, p.29-34.
Item 17. Links to empirical data: Evidence (e.g., quotes, field notes, text excerpts, photographs) to substantiate analytic findings.	p.16 – p.29
**Discussion**	
Item 18. Integration with prior work, implications, transferability, and contribution(s) to the field: Short summary of main findings, explanation of how findings and conclusions connect to, support, elaborate on, or challenge conclusions of earlier scholarship; discussion of scope of application/generalizability; identification of unique contribution(s) to scholarship in a discipline or field	Y - p.29-34.
Item 19. Limitations: Trustworthiness and limitations of findings	Y line 14 p.33 to line 12 p.34
**Other**	
Item 20. Conflicts of interest: Potential sources of influence or perceived influence on study conduct and conclusions; how these were managed.	Y p.5
Item 21. Funding: Sources of funding and other support; role of funders in data collection, interpretation, and reporting.	Y p.5

Appendix Two:

Consolidated criteria for reporting qualitative studies (COREQ): 32-item checklist (Tong et al., 2007).

Item	Description	Completed?
Domain 1: Research team and reflexivity		
Personal Characteristics		
1. Interviewer/facilitator	Which author/s conducted the interview or focus group?	Y p.11 line 23 to p.12 line 13
2. Credentials	What were the researcher's credentials? E.g. PhD, MD	Y p.11 line 23 to p.12 line 13
3. Occupation	What was their occupation at the time of the study?	Y p.11 line 23 to p.12 line 13
4. Gender	Was the researcher male or female?	Y p.11 line 23 to p.12 line 13
5. Experience and training	What experience or training did the researcher have?	Y p.11 line 23 to p.12 line 13
Relationship with participants		
6. Relationship established	Was a relationship established prior to study commencement?	Y p.11 line 23 to p.12 line 13
7. Participant knowledge of the interviewer	What did the participants know about the researcher? e.g. personal goals, reasons for doing the research	Y p.11 line 23 to p.12 line 13
8. Interviewer characteristics	What characteristics were reported about the interviewer/facilitator? e.g. Bias, assumptions, reasons and interests in the research topic	Y briefly p.11 line 23 to p.12 line 13 (and main analyst bias is considered in detail in SOM Section Three)
Domain 2: Study Design		
Theoretical Framework		
9. Methodological orientation and Theory	What methodological orientation was stated to underpin the study? e.g. grounded theory, discourse analysis, ethnography, phenomenology, content analysis	Y- Framework Method described line 17 p.14 to line 25 p.15 (and in detail in SOM Section 3)
Participant Selection		
10. Sampling	How were participants selected? e.g. purposive, convenience, consecutive, snowball	Y line 10 -14 p.11; line 9- 22 p.13
11. Method of approach	How were participants approached? e.g. face-to-face, telephone, mail, email	Y - line 15 - 20, p. 11
12. Sample size	How many participants were in the study?	Y – line 18 – 19, p.16
13. Non-participation	How many people refused to participate or dropped out? Reasons?	Y – line 16 – 24, p.16
Setting		
14. Setting of data collection	Where was the data collected? e.g. home, clinic, workplace	Y – line 21 to 23, p.11

15. Presence of non-participants	Was anyone else present besides the participants and researchers?	Y – line 24-25, p.11
16. Description of sample	What are the important characteristics of the sample? e.g. demographic data, date	Y – line 25 p.16 to line 15 p.17
Data Collection		
17. Interview guide	Were questions, prompts, guides provided by the authors? Was it pilot tested?	Y line 14 – 15, p.12
18. Repeat interviews	Were repeat interviews carried out? If yes, how many?	Y line 21 – 22, p.11
19. Audio/visual recording	Did the research use audio or visual recording to collect the data?	Y line 6 – 8, p.13
20. Field notes	Were field notes made during and/or after the interview or focus group?	Y line 10, p.13
21. Duration	What was the duration of the interviews or focus group?	Y line 21, p.11
22. Data saturation	Was data saturation discussed?	Y line 16-24, p.13; line 17-19, p.29.
23. Transcripts returned	Were transcripts returned to participants for comment and/or correction?	
Domain 3: Analysis and Findings		
Data Analysis		
24. Number of data coders	How many data coders coded the data?	Y – one coder, one supervisor, and then lived experience data checking
25. Description of the coding tree	Did authors provide a description of the coding tree?	Y, line 9-10, p.15; Table 2 p.45
26. Derivation of themes	Were themes identified in advance or derived from the data?	Y line 9 – 13, p. 15
27. Software	What software, if applicable, was used to manage the data?	Y line 14, p.15
28. Participant checking	Did participants provide feedback on the findings?	N but discussed line 4 - 8, p.34
Reporting		
29. Quotations presented	Were participant quotations presented to illustrate the themes / findings? Was each quotation identified? e.g. participant number	Y – throughout results
30. Data and findings consistent	Was there consistency between the data presented and the findings?	Y
31. Clarity of major themes	Were major themes clearly presented in the findings?	Y
32. Clarity of minor themes	Is there a description of diverse cases or discussion of minor themes?	Y (detailed focus on three less positive respondents to promote equipoise)
